# Redesigning navigational aids using virtual global landmarks to improve spatial knowledge retrieval

**DOI:** 10.1038/s41539-022-00132-z

**Published:** 2022-07-19

**Authors:** Jia Liu, Avinash Kumar Singh, Anna Wunderlich, Klaus Gramann, Chin-Teng Lin

**Affiliations:** 1 CIBCI Centre, Australian AI Institute, School of Computer Science, Faculty of Engineering and Information Technology, University of Technology Sydney, Ultimo, NSW Australia; 2 Biological Psychology and Neuroergonomics, Berlin Institute of Technology, Berlin, Germany

**Keywords:** Learning and memory, Cognitive neuroscience

## Abstract

Although beacon- and map-based spatial strategies are the default strategies for navigation activities, today’s navigational aids mostly follow a beacon-based design where one is provided with turn-by-turn instructions. Recent research, however, shows that our reliance on these navigational aids is causing a decline in our spatial skills. We are processing less of our surrounding environment and relying too heavily on the instructions given. To reverse this decline, we need to engage more in map-based learning, which encourages the user to process and integrate spatial knowledge into a cognitive map built to benefit flexible and independent spatial navigation behaviour. In an attempt to curb our loss of skills, we proposed a navigation assistant to support map-based learning during active navigation. Called the virtual global landmark (VGL) system, this augmented reality (AR) system is based on the kinds of techniques used in traditional orienteering. Specifically, a notable landmark is always present in the user’s sight, allowing the user to continuously compute where they are in relation to that specific location. The efficacy of the unit as a navigational aid was tested in an experiment with 27 students from the University of Technology Sydney via a comparison of brain dynamics and behaviour. From an analysis of behaviour and event-related spectral perturbation, we found that participants were encouraged to process more spatial information with a map-based strategy where a silhouette of the compass-like landmark was perpetually in view. As a result of this technique, they consistently navigated with greater efficiency and better accuracy.

## Introduction

The ability to navigate through a dynamic environment is considered to be a foundational skill for all organisms^1–3^. A wealth of studies on human history and animal habits demonstrate that spatial navigation skills can directly impact the evolution and survival of many species^3–5^. Spatial skills not only affect a person’s sense of independence and well-being, but they are also linked to improved brain functions, like increased neural connectivity as mental maps are formed^6–10^. A well-known study on the comparatively larger hippocampi of London cab drivers is a good example of how spatial navigation skills can manifest physically^11^. On the other hand, a decline in navigation skills might be a sign of decreasing neural functions. A recent study on aging highlights decreased navigation abilities in elderly populations^4^ and in individuals with both early-stage symptomatic Alzheimer’s disease and with preclinical Alzheimer’s disease displaying navigational deficits^12–14^.

Typically, individuals apply two fundamentally different strategies in navigational activities: a beacon-based strategy (also called ‘taxon’) and a map-based strategy (also called ‘locale’)^9,15,16^. The beacon-based strategy relies on an egocentric spatial system where the user heads directly toward a nearby goal. Similar to viewpoint-dependence common in turn-by-turn navigation, a beacon-based strategy will contain a list of locations, turns, and movements based on self-centred representations^9,17^. A well-known example of this strategy is the simple turn-by-turn instructions given by Google Maps. Alternatively, with map-based learning, individuals identify their position and navigate to a goal with respect to an array of landmarks. These landmarks can be some distance away, but the navigator usually has a constant spatial relationship to the landmark, akin to the spatial knowledge obtained from a map^9,16^. In a map-based strategy, representations are viewpoint-independent and located within a Euclidean system, yielding a space within a framework for relating objects or landmarks to each other independent of the observer^9^. Thus, though the map-based layout is more difficult and complicated for navigators to construct, it can build cognitive pictures in a highly flexible manner that efficiently guide behaviour when navigating, such as finding the shortest route to a destination. Numerous studies have proven the independence of these two strategies in humans with neuropsychological experiments^18,19^. The evidence shows that people tend to constantly prefer viewpoint-dependent or viewpoint-independent information while navigating. The implication being that people use only one specific strategy in different navigation environments^20–23^. Furthermore, studies also reveal an interaction between the beacon- and the map-based strategies in human spatial learning where participants switch strategies during experiments^16,24,25^. For example, participants switch from map-based learning to beacon-based learning in the presence of a visible goal^24,26^, and reverse the switch when the goal or the route is blocked^27^.

Current navigational aids mainly rely on the beaconing strategies that apply to turn-by-turn navigation instructions^28,29^, relegating map-based strategies to the side-lines. A simple example is instructing a patient in a hospital to follow a particular-coloured line from the entrance to a particular ward. More generally, the turn-by-turn instructions based on global positioning systems (GPS) have been widely used to plan routes. Almost everyone worldwide relies on this technology, either directly or indirectly in their everyday lives. Today, at least a dozen different navigation assistance services use GPS to provide reliable, efficient, and automatic orientation. However, these navigational aids ignore map-based strategies, which are fundamental to spatial learning. Cognitive maps are often formed through exploration at the most basic level, such as trial and error^1,9,27^. Building a map-like representation requires the integration of local and distant landmarks and their relative positions, as well as the routes connecting them^30,31^. The simple turn-by-turn instructions provided by a GPS assistance system do not require processing of environmental information and could thus negatively impact the construction of mental maps^32–34^.

It is therefore important to rethink how the next-generation of navigation assistance systems can provide navigation services while also support map-based learning. Similar to the role a compass plays in map reading, one of the features of map-based navigation is that it requires directional information as a solid reference of the direction an individual is heading in^9,35,36^. Distant landmarks, like city skylines or mountains, features that are visible from far away in contrast to local landmarks that are located in the immediate environment can serve as a reference direction and aid a navigator in maintaining accurate heading information^31,37^. For example, suppose a distant mountain sits northwest of a navigator. In this case, this mountain will remain a solid beacon in the northwest direction, no matter where the navigator is placed within the map space. Thus, as long as the mountain is visible, it can always be used by the navigator to determine their heading. However, in our modern era of urbanisation, the view of distant landmarks is blocked much of the time, especially as one approaches a city area. This restricted view increases the difficulty of map-based navigation, encouraging a beaconing-only strategy. Thus, inspired by the role of distant landmark in map-based navigation, we incorporated distant landmarks into a virtual heads-up display to provide a steady spatial reference that is visible at all times, even when the landmark is obscured in the real-world.

**Fig. 1:**
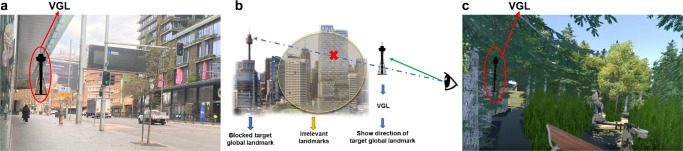
Design of the VGL system. **a** First person point-of-view during active navigation in an augmented reality environment. The global landmark is constantly present on the AR glasses and outlined in silhouette when obscured from view. **b** The working mechanism of the VGL system. As the selected reference of a global landmark is blocked by irrelevant landmarks, the VGL is presented to continuously indicate the direction of target global landmark. **c** First person point-of-view during active navigation in the fully virtual environment. Again, the global landmark is always present, even when obscured from view by other objects.

Figure 1a shows an augmented reality point-of-view for our proposed navigational aid system. Dubbed the virtual global landmark (VGL) system, the system provides the user with a view of a distant landmark as a steady reference 100% of the time, even when nearby cues would block the landmark’s view (see Fig. 1b). The main motivation for our study is to promote the map-based strategy in navigation. It is our intention that such a strategy will benefit cognitive exercise, especially the construction of cognitive maps^9^. In addition to fostering better retrieval of the spatial knowledge in a map frame, navigators will also be able to optimally plan routes with this strategy. In our VGL design, users will be able to maintain their sense of direction while always being aware of their own position relative to the VGL. This technique will enable the user to integrate other spatial locations relative to the VGL in the form of a cognitive map^9,27^. Thus, we predict that giving users enhanced landmark visibility should both encourage a map-based strategy and lead to efficient navigation.

To systematically investigate the efficacy of the system on map-based spatial learning from both behavioural and neural measures, we conducted an experiment involving a series of navigation tasks with 27 students from the University of Technology Sydney. For the neural measure, we used electroencephalography (EEG) to visualise the human brain’s dynamics in response to the activities. In the experiment, some participants were provided with VGLs while others were not and the EEG readings of the two groups were compared. EEG is a typically non-invasive electrophysiological monitoring method of collecting data on the brain’s dynamics with the electrodes placed along the scalp^38^. With the advantage of full mobility over traditional brain imaging technologies^39,40^, EEG has been one of the most popular neural measures to produce a reading of the brain’s spontaneous electrical activity in navigation studies^19,29,40^. For example, in the studies on the navigational strategies of reference frames, EEG power spectral modulation revealed the differences when individuals applied body-based or object-based reference frames^41–43^. In terms of research related to landmarks, studies have used event-related brain signals to investigate the effect of landmarks on spatial navigation through amplitude differences in the EEGs – an indicator believed to reflect the encoding and retrieval of spatial information^19,29,40^. In addition, as research continues to reveal the particular brain regions involved in spatial navigation, e.g. frontal and parietal corticals^44–48^, more studies on navigation have focused on activations in specific brain areas^49–51^. Thus, independent component analysis (ICA) has been broadly used to grab and separate different brain components for EEG signals^52,53^. Through an analysis based on frontal and parietal regions, the fronto-parietal network has been shown to involve viewpoint-independent learning during navigational activity^41,43,54^. Therefore, in our study, we evaluated event-related spectral perturbations (ERSP) originating in the frontal and parietal components through ICA analysis to investigate the brain dynamics associated with the specific navigational activity. We were interested to see whether brain dynamics in the parietal and frontal cortical areas would differ when participants were provided with solid compass information through the VGL during the navigation task as compared to when they navigated without in the same environment.

During the experiment, each participant was equipped with a Mobile Brain/Body Imaging (MoBI)^55–57^ system, which captures brain dynamics via EEG signals, and a head-mounted VR system to explore a medium-scale VR environment called “Sydney Park”. We also took notes about behavioural activity and asked each of the participants to complete a Santa Barbara Sense of Direction^58^ (SBSOD) test and a Perspective Taking/Spatial Orientation Task^59^ (PTSOT) before completing the trials as a control for individual differences in spatial abilities. Figure 1c shows the user’s view of Sydney Park. Sydney Park was specifically designed to offer a natural environment, similar to the Sydney Botanical Gardens, with visible local and distant landmarks^60^. Additionally, the scenario contained 11 local landmarks and three distant landmarks (including a lighthouse, the Sydney Opera House, and the Sydney Tower Eye) and other features to generate a realistic impression of the environment, such as paths, intersections, bushes, and trees. In this experimental scenario, participants were asked to follow a predefined route to explore Sydney Park. During the initial exploration phase, no VGLs were presented. The route was defined to balance participant exposure to local and distant landmarks. Following the exploration phase, participants performed wayfinding and pointing tasks to ascertain the spatial knowledge they had gleaned from the environment they had just learned. Twelve defined landmarks (11 local landmarks and one distant landmark) were potential targets for the pointing and wayfinding tasks. We tested two conditions in the wayfinding and pointing tasks within participants: navigation with and without VGL (called the VGL and non-VGL trials, respectively). In the non-VGL trials, participants conducted the spatial tasks based on the same local and distant landmarks they saw during the initial exploration phase only. In the VGL trials, in addition to the local and distant landmarks, participants were also able to see the VGLs as a steady compass reference. The pointing task asked participants to simply turn and point in the direction of a local landmark, which could not be seen from the test position. The wayfinding task required them to move toward that landmark – a task that involved route-planning based on a map representation of the environment^61–63^. Both the pointing and wayfinding tests are equally effective methods for assessing spatial learning and cognitive map building skills^64–66^. An example of a participant performing the exploration phase, together with the wayfinding and pointing tasks is presented in Videos 1 and 2 in the supplementary videos. Based on the results of a small pilot study we had conducted with a VGL system and directional arrows^67^, we hypothesised that with the aid of the VGL system, participants could be encouraged to engage a map-based strategy during navigation. As such, they would integrate more heading information into their cognitive maps and operate more efficiently during active navigational tasks than those without the aid of the VGL system.

## Results

Performance in the wayfinding and pointing task were assessed via one-way repeated-measures analyses of variance (ANOVA). Separate ANOVAs were computed for (i) response times in the pointing task; (ii) angular errors to targets in the pointing task; (iii) response times in the wayfinding task; and (iv) distance travelled in the wayfinding task. The trial condition (VGL and non-VGL trials) was entered as a within-subject factor. All variables were normally distributed (*p* > 0.05) and used Spearman’s rank-order correlation to assess the relationship between individual spatial ability factors (SBSOD and PTSOT scores) and the dependent measures. The Spearman’s correlations result between the individual’s spatial ability and the above measures are shown in Supplementary Fig. 1 of the supplementary information. Significantly correlated factors were entered as covariates ANCOVAs to control for the individual differences in spatial abilities. Additionally, gender was added as a between-subjects factor when calculating one-way repeated-measures ANOVAs. However, there was no significant interaction between gender and VGL conditions (non-VGL and VGL trials) for any of the measures. The test results of the between-subject effects are provided in Supplementary Methods of the supplementary information.

### Participants are more effective and show higher accuracy in wayfinding and pointing task with virtual global landmarks

**Fig. 2:**
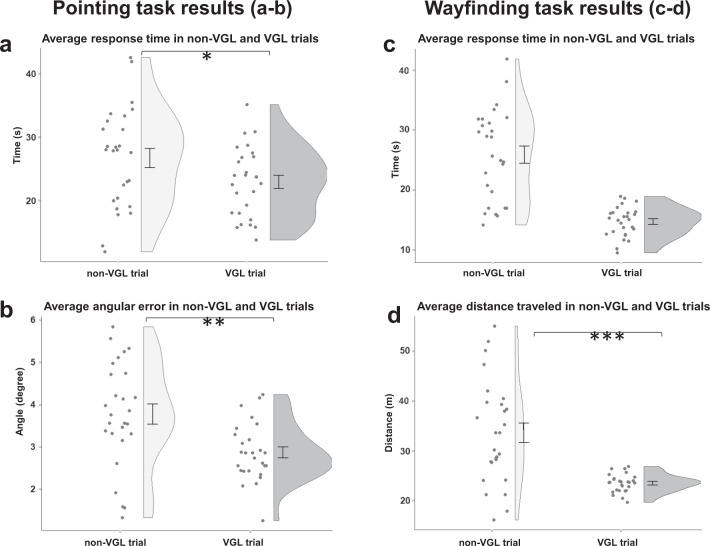
Behavioural results from the wayfinding and pointing tasks: with and without global landmarks visible. Each dot of the scatter plot indicates the average value for one subject. The results of the pointing task: **a** Average response time in seconds; and **b** Average angular error. The results of the wayfinding task: **c** Average response time; and **d** Average distance travelled. The black dots represent means, and the error bars indicate standard errors. *p < 0.05, **p < 0.01, ***p < 0.001.

To investigate spatial efficiency, when participants were aided with VGLs as solid compass information to support map-based strategy, we evaluated response times for trials with and without VGLs in the pointing and wayfinding tasks. In addition, we evaluated angular errors in the pointing task and distance travelled in the wayfinding task to assess the learning outcomes for different trial conditions. The results are shown in Fig. 2a–d.

As presented in Fig. 2a, b, participants demonstrated 31.8% lower angular errors and 16.5% faster response times with the aid of the VGL system than without, which indicated a higher accuracy in directional recognition and optimal use of time with VGLs. For the statistical detail, the ANOVA results of the pointing task revealed a statistically significant difference in response times between trials with and trials without augmented landmarks (*F*_1,26_ = 6.311, *p* = 0.019, partial *η*^2^ = 0.195) with shorter response times in VGL trials (*M* = 22.899, SE = 1.056) compared to trials without augmented landmarks (*M* = 26.673, SE = 1.521) with a difference of 3.774 s (95% CI, .686 to 6.86). In addition, a statistically significant difference in angular errors for the different trial conditions (*F*_1,25_ = 13.384, *p* = 0.001, partial *η*^2^ = 0.349) with less angular errors in augmented landmark trials (*M* = 2.866, SE = 0.115) than that in non-landmark trials, (*M* = 3.776, SE = 0.231) by .911 (95% CI, 0.410 to 1.411) degrees was observed. The SBSOD scores were included as a covariate in the ANCOVA for angular errors since a significant and positive correlation was shown for the angular errors and SBSOD values (rs(27) = 0.542, *p* = 0.004.

The results from the wayfinding task are shown in Fig. 2c, d. With the aid of a VGL, participants travelled a 43.0% shorter distance, which revealed optimal route-planning with VGLs. ANOVA showed a significant difference in the distances travelled (*F*_1,26_ = 26.939, *p* < 0.001, partial *η*^2^ = 0.509) with a shorter distance participants travelled in VGL trials (*M* = 23.501, SE = 0.343) than that travelled in non-VGL trials (*M* = 33.606, SE = 1.968) by 10.104 (95% CI, 6.103 to 14.106) metres. However, while entering SBSOD scores as a covariate, because of the significant negative correlation between the VGL trial and SBSOD test (rs(27) = –.471, *p* = 0.013), the ANOVA did not reveal any statistical difference between the response times for the two trial conditions (*F*_1,25_ = 1.269, *p* = 0.271, partial *η*^2^ = 0.048).

### A heatmap of local landmark fixation shows less fixation with the presentation of virtual global landmarks

**Fig. 3:**
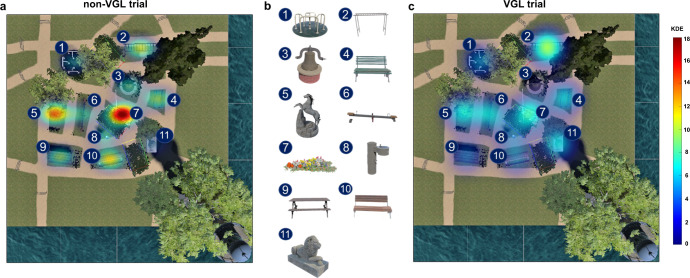
Heatmap of fixations on local landmarks during wayfinding and pointing tasks for VGL and non-VGL trials. The background of the heatmaps is the top view of the scenario and the trees surrounding the scenario have been removed for a clear view. **a** Heatmap of fixations on local landmarks during wayfinding and pointing tasks in non-VGL trials. **b** Map of local landmarks in the scenario. Label 1 to Label 11 represent a spinning wheel, monkey bars, a bell sculpture, a green bench, a horse sculpture, a seesaw, a parterre, a water fountain, a picnic table, a brown bench and a lion sculpture, respectively. **c** Heatmap of fixations on local landmarks during wayfinding and pointing tasks in VGL trials. The landmark label beside each landmark in the heatmaps is consistent with the respective label shown in **b**. The colour bar reflects kernel density estimation (KED) of fixations on each landmark.

To assess how frequently participants fixated on local landmarks during the pointing and wayfinding tasks, we generated heatmaps of visual stimuli taken from an eye tracker built inside the head-mounted goggle system (see Fig. 3). The heatmaps were generated from 156 trials of each landmark condition from all participants. More details on the fixation amounts for each local landmark are provided in Supplementary Fig. 2 of the supplementary information. Additionally, we calculated the number of each participant’s fixations for each landmark. For the analysis of the fixation heatmap, a 2 × 11 factorial repeated-measures ANOVA was computed with the within-subjects factor for trial condition (VGL and non-VGL) and landmark identity (1 to 11, consistent with the labels in Fig. 3).

As a comparison between the fixation on local landmarks in trials without and with VGLs, the results indicated that participants more often fixated on the local landmarks when they did not have a VGL to orient to, leading to a 144% higher number of fixations on local landmarks. The ANOVA result revealed significant main effects for both the trial condition (*F*_1,25_ = 68.721, *p* < 0.001, partial *η*^2^ = 0.733) and the landmark identity (*F*_10,250_ = 16.649, *p* < 0.001, partial *η*^2^ = 0.400). There was also a significant interaction effect between the two factors (*F*_10, 250_ = 11.590, *p* < 0.001, partial *η*^2^ = 0.317). The post hoc comparison results are presented in Supplementary Table 1 and Supplementary Table 2 of the supplementary information. Interestingly, the heatmap in Fig. 3a shows a higher number of fixations on landmark no. 7 compared to other landmarks in trials where no additional virtual landmark was presented. The post hoc comparison results (see Supplementary Table 2) revealed that landmark no. 7 drew significantly more fixations than other local landmarks when no additional VGL was present. This result may indicate the alternative compass-like information when no global orienting beacon was provided. Since landmark no. 7 was located in the centre of the scenario, this location may have been easier for the participant to remember as a central spatial reference for their mental representation of the environment.

### Mean spectral power changes in the theta, alpha and beta band of frontal and parietal regions

**Fig. 4:**
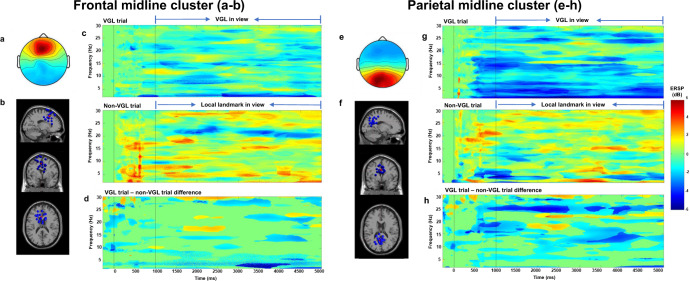
Frontal midline cluster (left, MNI coordinates *x* = −4, *y* = 19, and *z* = 43) and parietal midline cluster (right, MNI coordinates *x* = −4, *y* = −52, and *z* = 30). **a**, **e** Scalp maps for frontal (**a**) and parietal (**e**) midline clusters. **b**, **f** Equivalent dipole locations of independent components (in or near the frontal (**b**)/parietal (**f**) cortex) at the sagittal, coronal, and top view, respectively. **c**, **g** ERSPs in VGL and non-VGL trials from frontal (**c**) and parietal (**g**) midline clusters (first dotted lines at the 0 ms time point signify the onset of a trial, and the second dotted lines at the 1000 ms time point signify the onset of stimuli). **d**, **h** Significant differences between midline clusters in the two trial conditions (ERSPs of VGL trials minus the ERSPs of non-VGL trials) with *p* < 0.001 for the frontal (**c**) and parietal (**g**) cortices. For all ERSPs, non-significant points were masked with zero values in the mean ERSPs and are displayed in green. Significant differences with respect to baseline activity are displayed in red and blue for positive and negative deviations from the baseline activity, respectively.

Spectral fluctuations associated with the navigation tasks were evaluated in frontal and parietal regions through analyses of event-related spectral perturbations (ERSPs) of distinct frequency bands in clusters of independent component processes (ICs) derived from an independent component analysis (ICA)^68^, and subsequent equivalent dipole modelling. K-means clustering on dipole location of individual IC-processes resulted in a frontal cluster with its centroid located in or near the medial frontal gyrus (Brodmann area 8) containing 19 ICs from 15 participants, while the parietal cluster was located in or near the precuneus (Brodmann area 31) containing 18 ICs from 17 participants. Figure 4 shows the scalp maps (Fig. 4a, e), dipole location overlaid on a standard head model (Fig. 4b, f), and average ERSPs for the period from -1 to 5 seconds (Fig. 4a, e), for the frontal and parietal midline clusters time-locked to participants reaching one of the virtual checkpoints. The checkpoint map is illustrated in Fig. 6 of the Method section. The vertical line at 1000 ms indicates the onset of one kind of landmark in view. After 5000 ms, participants reached the next virtual checkpoint position (offset in ERSPs). In all trials, only one landmark of any kind was viewed before reaching the next checkpoint (i.e., a virtual global landmark in the VGL trial or a local landmark in the non-VGL trial). In total, there were 703 trials in the non-VGL condition and 1071 trials in the VGL condition for the frontal cluster. The analysis of ERSPs from the parietal cluster contained 769 trials in the non-VGL trial and 1074 trials in the VGL condition. For both clusters, the non-green pixels indicate differences in the ERSPs (Fig. 4d, h). Between the two types of trials, these differences were statistically significant at *p* < 0.001.

#### Average ERSPs in the frontal midline cluster

As presented in Fig. 4c, the average ERSPs for trials with VGLs revealed significant decreases in theta, alpha and beta activity, *p* < 0.001. In contrast, the ERSPs for trials with local landmarks showed significant increases in the same frequency bands, *p* < 0.001. During the VGL trials, a strong suppression started even a short time before the onset of VGL stimuli, especially for the theta and beta bands. The difference in ERSPs between conditions were statistically significant in these band activities, *p* < 0.001. The difference was especially pronounced in the theta and beta bands.

#### Average ERSPs in the parietal midline cluster

In the parietal cluster, for trials with VGLs the average ERSPs revealed strong decreases in theta, alpha, and beta band activity with onset of a trial. The suppression became stronger for a short time period before viewing the VGL. In the non-VGL trials, power increased significantly in the alpha and beta bands and decreased in the theta band (*p* < 0.001). Theta activity was suppressed around the onset of local landmarks, tapering back after 2 s. The differences in ERSPs between conditions was statistically significant for the alpha and beta bands over extended time periods.

## Discussion

This study systematically investigated the effects of VGLs on map-based spatial learning while individuals performed a physical navigation task. We used behavioural and neural measures to compare brain dynamics and behavioural activity in response to navigation tasks when participants were provided with a compass-like beacon through VGL as compared to task environments without. The learning outcomes in these two conditions was analysed using a pointing task^65,69^ followed by a wayfinding task^61,62,70^. The results revealed a significantly improved spatial orienting behaviour and optimal usages of time and route when using VGLs, as reflected in reduced landmark pointing errors and shorter response times, and shorter travelled distance for wayfinding. Moreover, local landmarks were used differently with and without the VGL system, as reflected in the participants’ gaze heatmap during the pointing and wayfinding tasks. The spectral perturbation patterns revealed a significant suppression in theta, alpha and beta band between views on local landmarks and VGLs. Overall, the VGL system offers users a reliable compass-like reference point, which supports them with the map-based learning strategy and enhances a better retrieval of spatial information and efficient navigation.

Pointing and wayfinding tasks are efficient and reliable measures that are broadly used to assess spatial knowledge acquisition^14,21,62,63,69–72^. In this study, we evaluated response times in pointing and wayfinding tasks to investigate spatial information recall efficiency, with or without VGLs. Additionally, we evaluated angular errors in a landmark pointing task and distances travelled in a wayfinding task to assess the accuracy of spatial knowledge retrieval.

We hypothesised that, with the aid of VGLs, participants would be encouraged to engage in a map-based strategy during navigation, integrate more heading information, and perform more efficiently through the map-based strategy while completing tasks. The results of the pointing task confirmed improved spatial performance for trials in which a VGL was visible. This result demonstrates an improvement in incidental spatial knowledge acquisition and navigation information perception for a new environment. As participants were not aware of the subsequent navigation tasks and the starting points of trials kept changing, they needed to point in the direction of targets based on their spatial memory, using a mental map based on their single exposure to a new environment. All target landmarks were only visible twice while navigating a fixed route in the exploration phase. Participants in the VGL condition pointed in the direction of landmarks with much greater precision, indicating that VGLs helped them retrieve their current position and orientation with higher precision. The fact that participants in the VGL trials took a shorter time to complete the pointing task suggests that they were able to retrieve the relevant spatial information faster and might have responded with higher certainty about landmark locations with the help of the virtual silhouettes. In summary, with the presence of a VGL, participants were able to estimate the direction of the target more precisely and efficiently, as reflected in the significant lower angular errors and less response times during pointing task, respectively. This result shows the capabilities of a map-based strategy on flexible direction recognition and the optimal time consumed.

Similarly, the wayfinding task results show that participants travelled a shorter distance when using the VGL system. That is to say, the VGL system helps participants to build a better representation of the shortest route from their current locations to the target. In other words, to successfully achieve the shortest travelled distance with different starting points, participants need to represent the direction of both target and the locations of self to flexibly determine a shortest route. This is likely because distant landmarks define a reference direction for an environment that allows for computing relative heading and bearing to other spatial locations in the same way a compass does^31^. Such solid references not only help participants to orient themselves, but also encourage a map-based strategy to build a mental map of new environments^37,73^. That said, there was no significant difference in response times for the wayfinding task between conditions with or without virtual landmarks. This might have been caused by participants continuously checking their progress against the virtual landmarks to ensure they were going in the correct direction using the shortest route, which may have resulted in comparable response times with the condition providing no virtual landmarks that required longer orientation times. Overall, these findings support the idea that distant landmarks strongly impact spatial knowledge retrieval by forming a reliable reference point for participants, allowing for the integration of other spatial locations in a map-based representation.

Importantly, for this study, we applied an immersive virtual reality (VR) protocol^40,74,75^ and provided a nearly natural experimental environment for this study. Previous studies using stationary experimental protocols that did not allow for movement of participants revealed differences in wayfinding performance when comparing virtual environments without natural movements and real-world environments with free locomotion^76^ However, there were significant correlations between the performances in the two types of environment^77^. In contrast, a study by Pastel and colleagues (2020) on spatial orientation in VR found that the performance of human navigators when actively moving in the virtual environment can be compared to the performance in real environments^60^. Our study allowed free movement through the virtual environment, similar to real-world navigation. By moving freely, participants could explore and sense their environment using all their natural senses, including visual, vestibular and proprioceptive feedback about changes in spatial location and orientation. Therefore, the impact of a VGL system on spatial knowledge retrieval as shown in the present VR environment may also improve the same ability in users in the real-world.

In addition, our heatmaps of fixation activities showed that participants searched significantly less for local landmarks when they were shown steady distant landmarks through VGLs. This may be because the distant landmarks provided participants with a more certain sense of orientation. Instead of blindly searching the environment for clues, they had a sense of how to get to the target. Interestingly, a significant higher fixation on local landmarks in trials without distant landmarks may indicate a navigation strategy by participants that, while lacking a global spatial reference direction, intend to refer a similar beacon to be a compass-like reference from the most centrally located local landmark that connects other locations in the environment.

Many studies suggest the parietal and frontal cortex play an important role in navigation and the integration of multimodal information as an important input to the spatial orientation system^49,78–81^. Besides, the fronto-parietal network also involves the viewpoint-independent and viewpoint-dependent reference proclivities during navigational activity in humans^41,43,54^. For this reason, we focused on the frontal and parietal regions when investigating how the brain responded to the wayfinding and pointing tasks. The results show significant changes in the EEG power modulations in the alpha (8–14 Hz), beta (15–30 Hz), delta and theta (<8 Hz) bands of the parietal and the frontal cluster.

Desynchronizations in the alpha band were observed with different temporal dynamics, as shown in Fig. 4c, g. Alpha suppression can be associated with increased information processing in the parietal cortex^82^. In addition, alpha oscillation in the parietal cortex could also relate to spatial information processing and navigation^83,84^. This would suggest an increase in the demand for attention and semantic memory, leading to a selective suppression of alpha band activity. This interpretation implies that the presence of VGLs leads to an increase in visuo-spatial processing and an integration of ones’ own position and orientation with respect to the environment. Furthermore, the strong alpha blocking before and during heading changes in or near the parietal and retrosplenial cortex is revealed by studies as an activity pattern underlying the translation of spatial information from a viewpoint-independent reference frame into a viewpoint-dependent reference frame and vice versa during navigation^41,42^. The strong parietal alpha blocking with a VGL in view likely reflects the integration of the egocentrically perceived distant landmark into a viewpoint-independent reference frame. The translation of spatial reference information may suggest participants integrated other spatial locations relative to the VGL when planning a route to the target, and thereby be supported with map-based strategy during spatial knowledge processing. The difference in ERSPs between the two types of trials also revealed a strong desynchronization in the delta and theta bands in the frontal cluster. It has been widely demonstrated that increased frontal theta activity reflects increased mental effort during navigation tasks^84,85^. Frontal theta activity in humans was also reported to increase with memory load^86^. Continuously increasing theta power in trials without virtual landmark support the assumption that participants exerted more effort to complete the wayfinding task using real landmarks alone. Owing to a lack of a continuous orienting beacon, participants needed to remember more intermediate local landmarks resulting in an increased memory demand.

Concerning beta band power, our analysis of the ERSPs in the wayfinding task shows a strong desynchronization of the beta band in both the parietal and the frontal cluster. Beta power is known to be associated with sensorimotor tasks^87^. It decreases when there is a change in movement and increases when movement has to be voluntarily suppressed^88–90^. Regarding the beta activity in VGL trials, the suppression of beta oscillations occurred shortly after trial onset and decreased continuously after VGLs were displayed. In trials without distant landmarks, beta power increased with the onset of the trial and was slightly suppressed shortly after the local landmark was in view. The ERSPs of condition differences revealed a statistically significant suppression in the VGL trials compared to that in the non-VGL trial. This result may imply a maintained orientation was achieved when participants were supported by VGL to plan a route for target in the wayfinding task. As revealed by the continuous beta suppression, participants might have been ready to navigate faster in the VGL trials. Compared to the trial with distant landmarks, the results of trials without the virtual orienting beacons might indicate a short time period with orientation loss when no local landmarks were in view during the wayfinding task. Only after seeing local landmarks did the participants gain directional information and start the movement. This result supports the assumption that participants were able to maintain their orientation in an environment by forming and encoding the relationships among multiple spatial stimuli through the steady presentation of distant landmarks.

Altogether, our findings suggest that a VGL system can encourage and help participants to process more spatial information for their surroundings compared to navigating based on local landmarks only. By enhancing the range of relevant viewpoint-independent cues by displaying a distant landmark in the environment, VGL systems can improve the integration of viewpoint-dependent and viewpoint-independent spatial information and encourage a map-based strategy. This could be a potential solution for the application of cognitive navigation assistance systems and navigation aids that aim to support spatial learning as opposed to merely showing us the way. For example, a potential application of the VGL system could be using virtual global landmarks in a next-generation navigation system (e.g., AR navigation system) with other navigation aids such as directional arrows and turn-by-turn instructions, to maintain navigation support, as well as encouraging users to process their spatial surroundings. Additionally, VGL systems have the potential to support elderly or early Alzheimer’s patients with specific impairments in allocentric navigation. In our future work, we will continue studying the VGL’s effect on spatial learning with a long-term knowledge acquisition perspective.

## Methods

### Participants

The experiment involved 27 participants: 9 females and 18 males (see Table 1 for demographic information). Before participating in the study, the experimental procedure was explained, and each participant provided informed consent. The Human Research Ethics Committee (HREC) of the University of Technology Sydney (UTS) also reviewed the protocols and issued their approval (ETH17-2095). All trials were conducted in the UTS Tech Lab. None of the participants reported a history of psychological disorders, which could have affected the experimental results. To control for individual differences in spatial abilities, we administered a SBSOD test^58^ and a PTSOT test^59^ prior to conducting the experiment.

**Table 1:** Participant demographics and average orienting ability test scores at the initial testing.

Women/men	Age^a^ (years)	SBSOD^a^	PTSOT^a^
9/18	28.19(±5.33)	0.67(±0.17)	26.31(±17.11)

^a^Standard deviations are shown in parentheses.

### The VGL system setup

To serve as a stable reference for the direction of specific locations without disturbing the overall environment, the VGLs were displayed as transparent, 2-dimensional silhouettes of the real landmark. VGLs were displayed at a location that resembled the distance and height as other local landmarks in the scenario to avoid uncomfortable eye movements. These were intended to provide effective spatial information on a head-mounted VR to enhance the spatial learning opportunities and abilities of users^67^. With the help of these virtual landmarks, users were subconsciously encouraged to continuously compute directions from a particular location. The silhouettes were projected by the VR in the direction of the landmark within the participant’s sightline. Whether walking or turning, as long as the participant looked in that direction, they were able to see either see the real landmark or the silhouette if it was blocked by another object in their surroundings. There were three distant landmarks in the Sydney Park scenario: a lighthouse, the Sydney Opera House, and the Sydney Tower Eye.

### VR and EEG setup

**Fig. 5:**
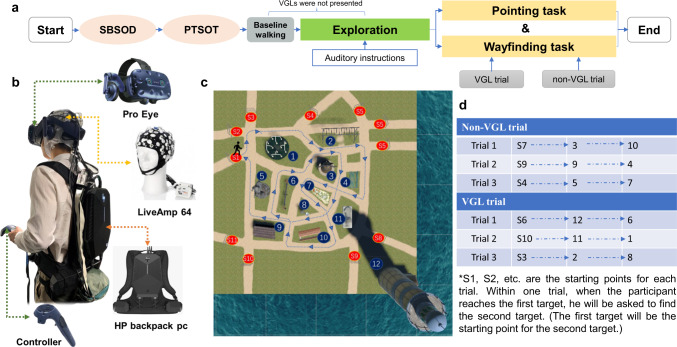
Experiment procedure. **a** Overview of the experimental procedure design. First, the participants completed a Santa Barbara Sense of Direction questionnaire about their sense of direction and a Perspective Taking/Spatial Orientation Task, which assesses spatial orienting ability. They were then asked to walk in a square meadow for five minutes. Next, each subject started walking through Sydney Park along a fixed route with auditory instructions. Global landmarks were not displayed during this exploration phase. Last, the pointing and wayfinding tasks were conducted. **b** The gear setup for each participant. During the tasks in the VR environment, participants were wearing a 64-channel EEG cap covered by the VR headset with an HP backpack PC on their back and were holding a controller. **c** A fixed route map of exploration Auditory instructions were used to guide participants through the route. The plants and trees inside and surrounding the scenario were removed for a clear view of the path. **d** Trial settings for wayfinding and pointing tasks. For each landmark target, the participant was asked to point to the target (pointing task) before moving toward it (wayfinding task). The number labels for each trial are consistent with the labels in **c**.

Figure 5b provides an overview of the setup for participants. The Sydney Park scenario was based on VR but imitates the real environment of the Sydney Botanical Gardens. The scenario was fully immersive so as to hold the participants’ attention during the duration of the navigation task experiments. We used the HTC’s Vive Pro eye headset with an embedded Tobii eye tracker. The Vive Pro eye uses a dual OLED 3.5” diagonal display with a resolution of 1440 × 1600 pixels per eye (2880 × 1600 pixels combined) and a refresh rate of 90 Hz, as reported by HTC. The participant’s head position was principally tracked with embedded inertial measurement units, while an external lighthouse tracking system cleared the common tracking drift with a 60 Hz update rate. We tracked the eye activity of participants using the Tobii eye tracker at a sampling rate of 120 Hz.

The EEG data were recorded continuously using Brain Vision’s LiveAmp 64 system (Brain Products, Gilching, Germany) using 64 active electrodes mounted on an elastic cap. The electrodes were positioned according to an extended 10–20 system^91^. The EEG signals were referenced to the electrode located at FCz and the impedance of all sensors was kept below 5 kΩ. EEG events were created when the participants’ fixated on the surface of a defined landmark, both real and virtual. All data streams from the EEG cap, eye tracker and head-mounted display were synchronised with Lab Streaming Layer (LSL).

### The Sydney Park scenario

Sydney Park was created in Unity 2018.3.5f1 (Unity Technologies, USA). Figure 5c shows a birds-eye view of the scenario (with plants and trees inside and surrounding the scenario have been removed for a clearer view). Two sides of the scenario were extended with only the sea, and a lighthouse positioned in the corner next to the ocean (labelled no. 12 in Fig. 5c). Of the remaining two sides, one had a view of the Sydney Opera House and the other had a view of the Sydney Tower Eye, similar to the views from the Royal Botanical Gardens.

**Fig. 6:**
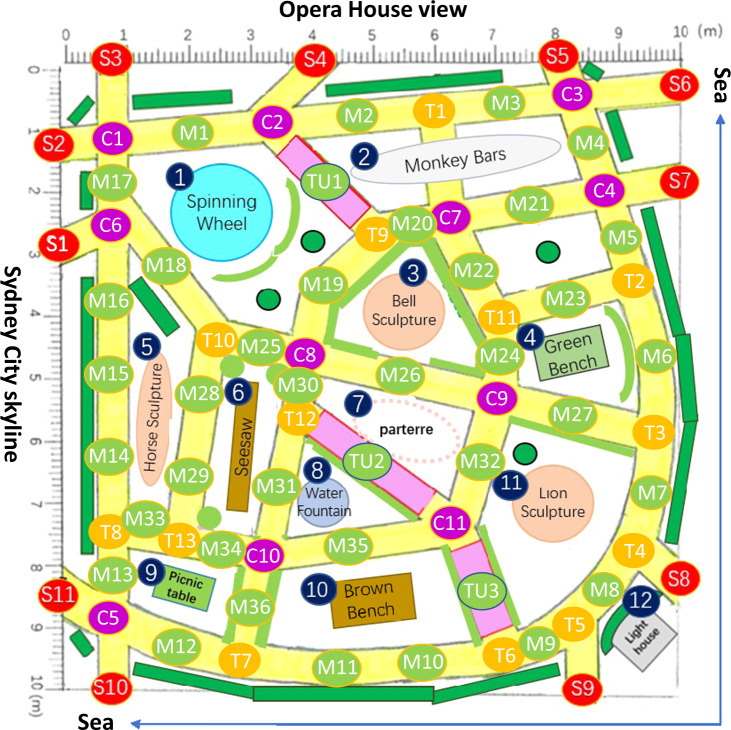
Checkpoint map. The red checkpoints define the starting points; the green checkpoints are those in the middle of one path; the purple checkpoints appear at crossroads; and the yellow checkpoints represent a T intersection. The background map is a sketch of the top view of the “Sydney Park” scenario.

The Sydney Park environment consists of 11 local landmarks (labelled no. 1 to no. 11 in Fig. 3b) and 3 distant landmarks (including a lighthouse, the Sydney Opera House and the Sydney Tower Eye), in combination with paths, intersections, bushes, trees, etc. To control the visibility proportion of all distant landmarks, we used background plants, e.g., trees, to ensure distant landmarks were obscured. The visible status for all distant landmarks was checked at each checkpoint (the checkpoint map is shown in Fig. 6). Overall, distant landmarks were blocked from view for nearly 60% of the time during the experiment.

### Experiment procedure

We conducted a pre-test (SBSOD^58^, PTSOT^59^) on all participants to assess their individual spatial abilities before starting. The participants then explored the Sydney Park scenario along a predefined route as shown in Fig. 5c. Afterwards, they performed two specific navigational tasks: one pointing task and one wayfinding task. All the tasks were conducted inside the Sydney Park scenario and involved active navigation, including physical walking. An overview of the experimental procedure is shown in Fig. 5a.

#### Pre-test

In this phase, we assessed the spatial ability of the individual participants before they performed exploratory and navigational tasks in the VR scenario. The participants were not aware of the following experimental procedure while completing this phase. Every participant completed the SBSOD questionnaire and a PTSOT test. As a subjective measure of individual sense of direction, SBSOD is a standardised self-report scale of environmental spatial ability, including 15 items^58^. The PTSOT, a questionnaire for evaluating spatial orientation ability, requires the participants to imagine themselves in a different orientation within an environment in order to indicate the direction of a target object relative to oneself and to other objects from the imagined perspective^59^.

#### Exploration phase

First, each participant first had five minutes to walk around inside a meadow area in the VR environment. Next, they started walking through the Sydney Park scenario along a fixed, predefined route with the assistance of auditory instructions (see Fig. 5c and Video 1 in the supplementary videos). This was intended to help and standardise how participants explored the environment. All target landmarks involved in the following tasks were passed just twice while navigating the fixed route. Virtual global landmarks were not displayed during the exploration phase.

#### Pointing and wayfinding tasks

In the next phase of the experiment, the participants were given two navigation tasks: pointing and wayfinding. They performed the pointing task while holding a controller, as shown in Fig. 5b. At a predefined starting point, the participant was asked to point to the centre of the target landmark. When pointing, they were only allowed to stand in place and rotate; walking was strictly prohibited to ensure the target could not be seen. To confirm the direction, the participant had to “lock in” their point by pressing a button on the controller while pointing with their other hand. Next, the participant was asked to find that same target in a wayfinding task. At this stage, the participant was allowed to walk freely around Sydney Park until they reached their target location. When participants conducted the pointing and wayfinding task, the distant landmarks were not visible from the starting points. In other words, participants could not refer to distant landmarks for the pointing task. In addition, for the wayfinding task, distant landmarks obscured almost 60% of the scenario, and thus participants could not always refer to distant landmarks. Under these conditions, we separated trials with virtual global landmarks and trials without them. In non-VGL trials, as described above, participants could not refer to distant landmark while in the VGL trials, distant landmarks were always visible either through direct visual contact of through a virtual global landmark. A total of six trials were completed with each participant, three trials under each landmark condition (with or without VGL). Within each navigation task, there were two landmark targets, and both were visited in sequence: i.e., pointing to Landmark 1; walking to Landmark 1; pointing from Landmark 1 to Landmark 2; then walking from Landmark 1 to Landmark 2. Figure 5d shows a map of all the start points and target landmarks. An example of one participant performing these tasks provided in Video 2 in the supplementary videos.

### Eye information analysis

We captured eye movements and other information separately for each eye and for each trial during the pointing and wayfinding tasks. All raw eye data were imported into MATLAB R2019b (MathWorks Inc., USA) for analysis. To assess when and for how long participants fixed their gaze on local landmarks, we calculated the total number of fixations for each trial type. The heatmap in Fig. 3a, c shows the fixations plotted with the *surf* function. We applied the *gkde* MATLAB tool^92^ to implement a Gaussian kernel density estimation of fixation.

### EEG analysis

#### Pre-processing

All raw EEG data were imported into MATLAB version 2018a (MathWorks Inc., USA) using the EEGLAB toolbox version 2020.0^53^ for further processing. For each participant’s raw data, we first checked the data quality by visual inspection. Of the 27 participants, data for two participants were excluded due to poor EEG quality with discontinuous signals. The raw data for the remaining 25 participants were first bandpass filtered from 1 Hz to 100 Hz and downsampled to 250 Hz. Then, data from each single-task were merged into one large EEG dataset for the following pre-processing steps. Line noise (50 Hz) and associated harmonics were removed using the *cleanline* function. Subsequently, dead channels were removed (threshold = 5 seconds) were removed using the *clean_flatlines* function in EEGLAB. Noisy channels were rejected with the *clean_channels* function. 4.62 ± 3.02 channels from all 64 channels were removed. All missing EEG channels were interpolated by spherical splines before re-referencing to the average of all channels. Noisy data in the time domain were removed through automatic continuous data cleaning with *pop_rejcont* function. We used the hamming taper window with a window length of 0.5 s and an overlap of 0.25 s based on the spectrum (threshold = 10 dB) to clean the continuous data of frequency range from 1 Hz to 100 Hz. On average, 42.70% ± 14.84% of the data in the time domain were removed. The data were then submitted to adaptive mixed independent component analysis (AMICA)^68^ to decompose the data into a series of statistically maximally independent components (ICs). For EEG analysis, the independent component analysis (ICA) method has been widely used in the EEG research community to remove non-brain noise in the data, such as a blink, muscle movement, and line noise^52,53^. Here, we took the AMICA approach to separate the brain sources from the non-brain signals and grab brain components in specific brain areas. As one of the key features, the source density models of AMICA are adapted using a mixture of the Generalised Gaussian density model, resulting in a good fit between the density model and the actual density of the sources being estimated. An example of raw EEG signals and ICA result from one participant are shown in Supplementary Figure 3 of the supplementary information. The equivalent dipole model of each independent component was computed using a boundary element head model as implemented in EEGLAB’s DIFIT2 routines, including setting model and preferences, grid scanning and non-liner interactive fitting^93^. Last, the spatial filter and dipole models were copied back to the pre-processed but uncleaned EEG single-task data (no cleaning in the time domain) for further analysis. The pipeline code for pre-processing is provided in Supplementary Methods of the supplementary information.

#### Trial extraction

The cleaned data for the wayfinding task were extracted with a time window of [–1 7] s. A baseline of [–1 0] s was applied for each epoch to calculate significant differences with respect to baseline activity. This way, only the specific stimulus-related activity in the ERSP was investigated. The onset and offset events were generated based on checkpoints (see Fig. 6). For each single period between two checkpoints (on the virtual global landmarks in the VGL trials or the local landmarks in the non-VGL trials), the onset event was generated at the first checkpoint, and an offset event was defined at the second checkpoint. Bad epochs were detected and removed based on component activities using the *autorej* function. On average, 0.15% ± 0.74% epochs of the non-VGL condition were removed; none of the distant landmarks were detected as bad epochs.

#### Independent component clustering

The independent components from all participants were first selected with less than 15% residual variation of the equivalent dipole model, and the components with an equivalent dipole model located outside the head sphere were removed. In other words, except brain components, all other components, including muscle, eye, heart were removed. Then, the selected components were clustered using K-means clustering in EEGLAB. To avoid the “double dipping” problem^94,95^, only dipole locations were included as the measure for clustering. The components from at least 60% of participants were grouped after this process. For the ERSP analysis, we focused on the clusters of components located in or near the parietal cortex and frontal cortex to find spatial navigation-related^44–48^ neural dynamics in the VGL and non-VGL trials. We used the Talairach client tool^96,97^ to evaluate the nearest grey matter of dipole locations from the targeted cluster centroid and clustered components. Additionally, to visualise the effect of cleaning our data with the removal of non-brain ICs, we have analysed our baseline data for standing and walking at the frontal and parietal clusters. During the baseline phase, the participants stood still on a blank meadow area (no visual stimuli) in the VR environment for one minute and walked on the meadow for another four minutes. The ERSPs for the baseline standing and walking are shown in Supplementary Fig. 4 of supplementary information.

#### Event-related spectral perturbation (ERSP) and statistics

We first computed the ERSPs at the single independent component level based on the cluster of interest, then averaged them at the participant level, and finally at the group level. ERSPs were plotted for each independent component with the *newtimef* function and linearly time-warped to epoch lengths of [–1 5] s. The time-frequency data of all independent components from the same participant were averaged. Then, the ERSPs of all participants were averaged for the final ERSPs at the group level. Significant differences from the baseline activity are displayed in red for positive deviations, blue for negative deviations, and green for non-significant differences. We determined conditional differences using the *newtimef* function with a statistical threshold of *p* < 0.001 as the two-tailed permutation significance probability level for all selected independent components. In the supplementary information, we provided a global view of our processing steps for EEG analysis in Supplementary Fig. 5.

### Statistical analysis

The statistical analyses were conducted using SPSS Statistics 26 (International Business Machines Corporation (IBM) Analytics, Armonk, USA). Data visualisations were created with the *ggplot* function of R^98^ (RStudio Inc, USA). We computed one-way repeated-measures ANOVAs^99,100^ for the within-subject factor trial by type (VGL and non-VGL) for the wayfinding and pointing tasks and for eye activity. The dependent values for this wayfinding analysis were the response time (time spent reaching the target) and the distance travelled by the participant. For the pointing task, the dependent values included the response times and the angular errors to the centre of the target. For the analysis of the fixation heatmap, a 2 × 11 factorial repeated-measures ANOVA was computed with the within-subjects factor virtual global landmark condition (VGL and non-VGL) and landmark identity (1 to 11, consistent with the labels in Fig. 3). Each measure was calculated separately for the VGL and non-VGL trials.

For all measures, we first explored the data to check if outliers existed for the within-subject factor computations. All outliers inspected by boxplots for values greater than 1.5 box lengths from the edge of the box were removed. We then calculated two mean values for each participant: one for the VGL trials and the other for the non-VGL trials. The mean values were then inspected again, and outliers were interpolated with the median value. In addition, we ran a Shapiro-Wilk test to determine whether the mean values for both types of trials were normally distributed for each level of the within-subject factor trial type (VGL and non-VGL). We used Spearman’s rank-order correlation to assess the relationship between individual spatial ability factors (SBSOD and PTSOT scores) and all measures. The Spearman’s correlations result is shown in the Supplementary Fig. 1 of supplementary information. With the significantly correlated factors, we then used the scores from these tests as covariates to assess how much the participants’ inherent, subjective sense of direction and orientating ability affected their completion of the navigation tasks.

### Reporting summary

Further information on research design is available in the Nature Research Reporting Summary linked to this article.

## Supplementary information

Supplementary Information

Reporting Summary

Supplementary Video 1 of exploration

Supplementary Video 2 of Pointing and Way Finding Task

## Data Availability

The data that support the findings of this study are available upon request from the corresponding author.

## Code availability

All custom MATLAB code used in this study is available upon request from the corresponding author.
