# Supplementary material for: Redesigning navigational aids using virtual global landmarks to improve spatial knowledge retrieval
Source: NPJ Sci Learn. 2022 Jul 19;7:17. doi: 10.1038/s41539-022-00132-z (PMC9296625; doi:10.1038/s41539-022-00132-z)
Supplement: Supplementary file 1 — Supplementary Information [file 41539_2022_132_MOESM1_ESM.docx]

# Supplementary for the manuscript of

# “Redesigning Navigational Aids using Virtual Global Landmarks to Improve Spatial Knowledge Retrieval”

Jia Liu^1^, Avinash Kumar Singh^1^, Anna Wunderwich^2^, Klaus Gramann^12^ and Chin-Teng Lin^1^

^1^ CIBCI Centre, Australian AI Institute, School of Computer Science, Faculty of Engineering and Information Technology, University of Technology Sydney, Australia; ^2^Biological Psychology and Neuroergonomics, Berlin Institute of Technology, Berlin, Germany

This supplementary material supports the information presented in the manuscript. The additional evidence provided here includes:

Supplementary Figures

- Correlation results of the individual spatial ability factors (SBSOD and PTSOT scores) and the dependent measures
- Gaze information for the heatmaps
- Example of raw EEG signals and ICA result
- ERSPs in frontal and parietal clusters for baseline standing and walking
- An EEG processing pipeline for the EEG analysis section

Supplementary Tables

- The post hoc comparison results for trial conditions (non-VGL and VGL)
- The post hoc comparison results for local landmarks

Supplementary Methods

- The effects results of gender as the between-subjects factor of ANOVAs
- The post hoc comparison results of trial type and landmarks for local landmark fixations in non-VGL and VGL trials
- EEG pre-processing pipeline code

## **Supplementary Figures**

*Correlation results of the individual spatial ability factors and dependent measures.* To control for individual difference in spatial ability, we used Spearman's rank-order correlations to assess the relationships between the individual spatial ability factors (SBSOD and PTSOT scores) and all measures. The results for each trial are shown in Figure S1. With significantly correlated factors, we used the individual spatial ability factors as a covariate to assess how much the participants’ inherent, subjective sense of direction and orientating ability affected their completion of the tasks.


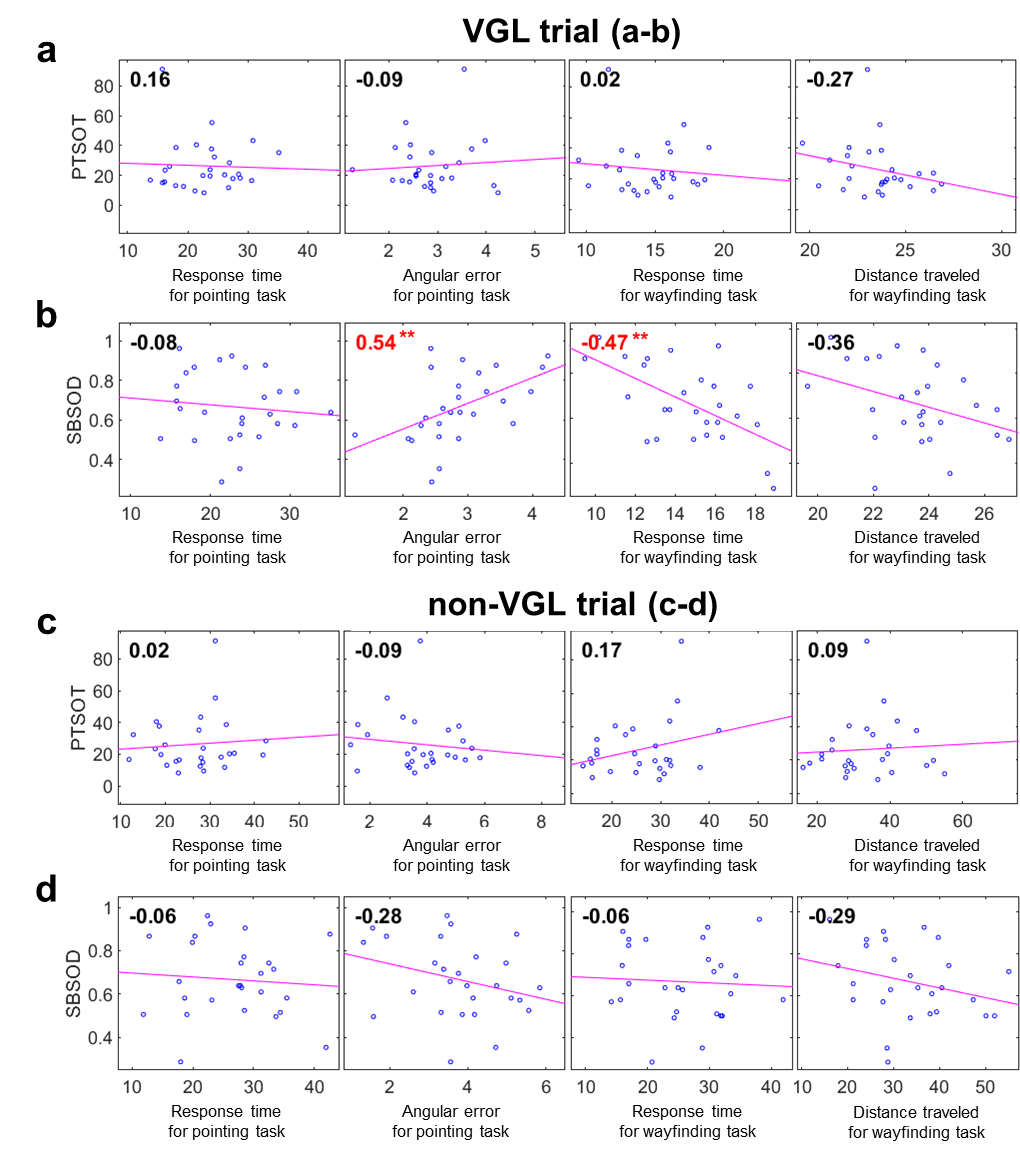


**Supplementary Figure 1: Correlation result of individual spatial ability factors and dependent measures.** (a-b) Measures correlated with PTSOT (a) and SBSOD (b) for VGL trial, respectively. (c-d) Measures correlated with PTSOT (c) and SBSOD (d) for non-VGL trial, respectively. Each figure shows the computed correlation coefficient with the number in the top left corner (red color indicated for statistical significance; ** indicated for *p* <.01.

*Landmark fixation information for heatmaps.* How much participants fixated on local landmarks during the experiment was calculated based on the output from the eye tracker. We used this data as input for the heatmaps in Figure 3(a, c). The heatmaps were plotted using the surf function in MathWorks MATLAB R2019b. To estimate the amount of fixation, we used the *gkde*^1^ tool to calculate the bivariant Gaussian kernel density.


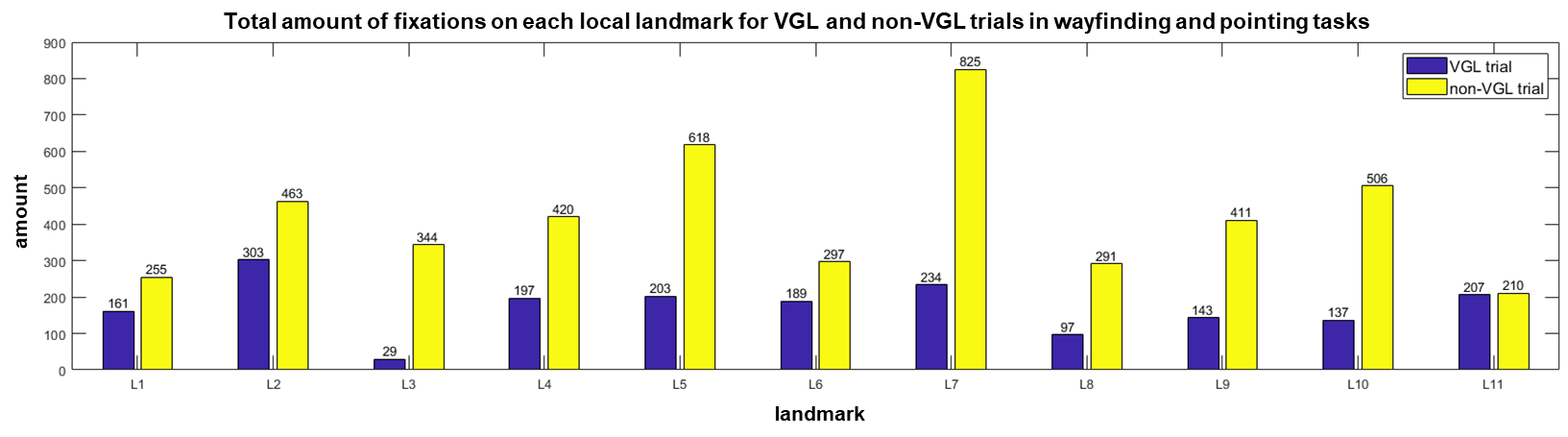


**Supplementary Figure 2: Total amount of fixations on each local landmark during the wayfinding and pointing tasks (both trials).** The number above each bar indicates the exact value. The number of landmarks is consistent with the label in Figure 4 (b).

*Example of raw EEG signals and ICA result.* This an example of raw EEG signals and ICA result from one participant.

**
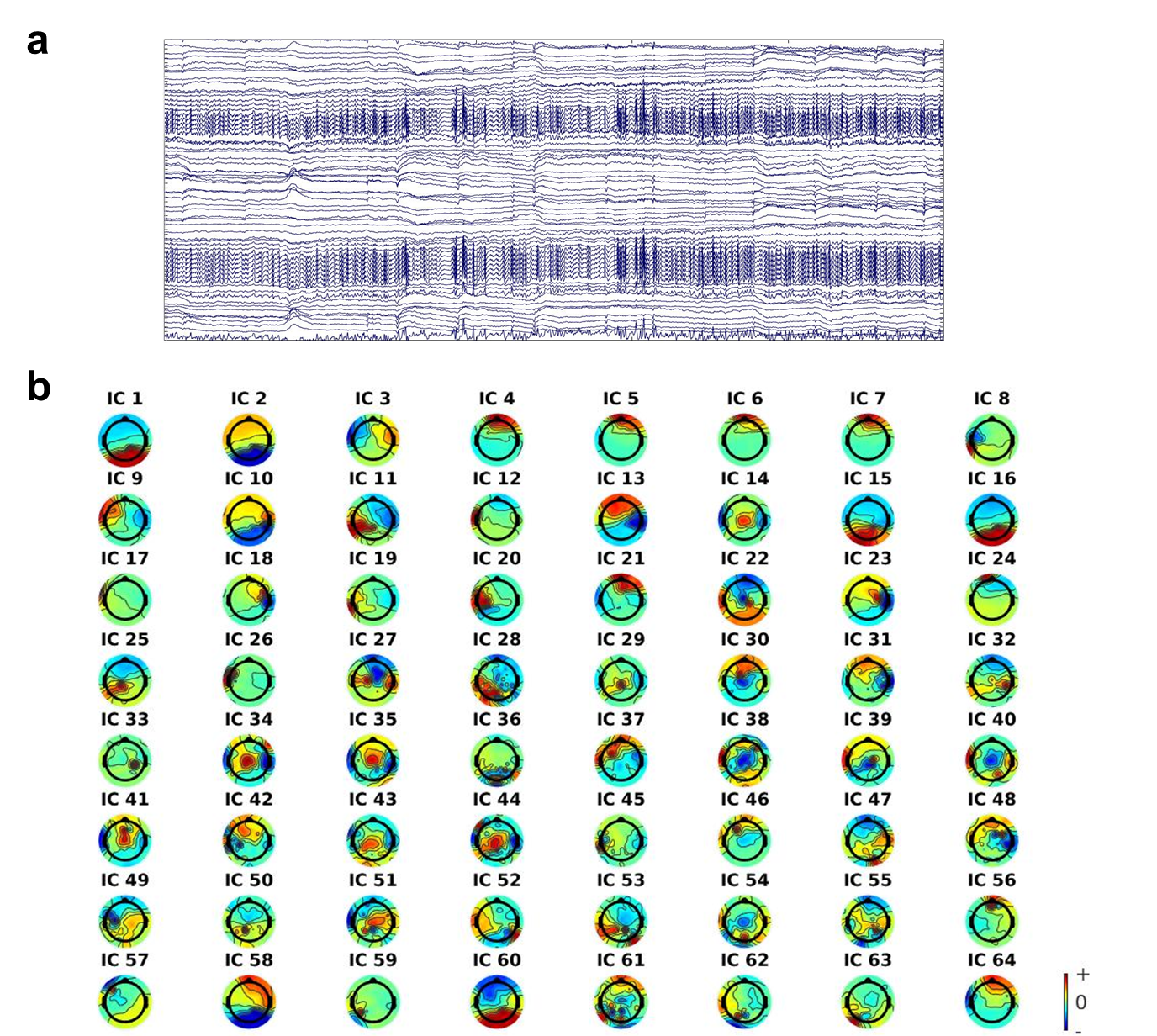
**

**Supplementary Figure 3: Example of raw EEG signals and ICA result from one participant.** (a) Raw EEG signals. Each line represents one EEG channel and total 64 channels are recorded. (b) Scalp maps for all the independent components. These components were obtained from the independent component analysis (ICA) of the 64 channels raw EEG data in (a). ICA helped to unmix the multi-channel EEG data into a sum of linearly independent, spatially fixed cortical sources.

*ERSPs in frontal and parietal clusters for baseline standing and walking.* To visualize cleaning effect on other components except brain one, we have analyzed our baseline data for standing and walking at the frontal and parietal clusters. During the baseline phase, the participants were standing still on a blank meadow area (no visual stimuli) in the VR environment for one minute and walking on the meadow for another four minutes.

**
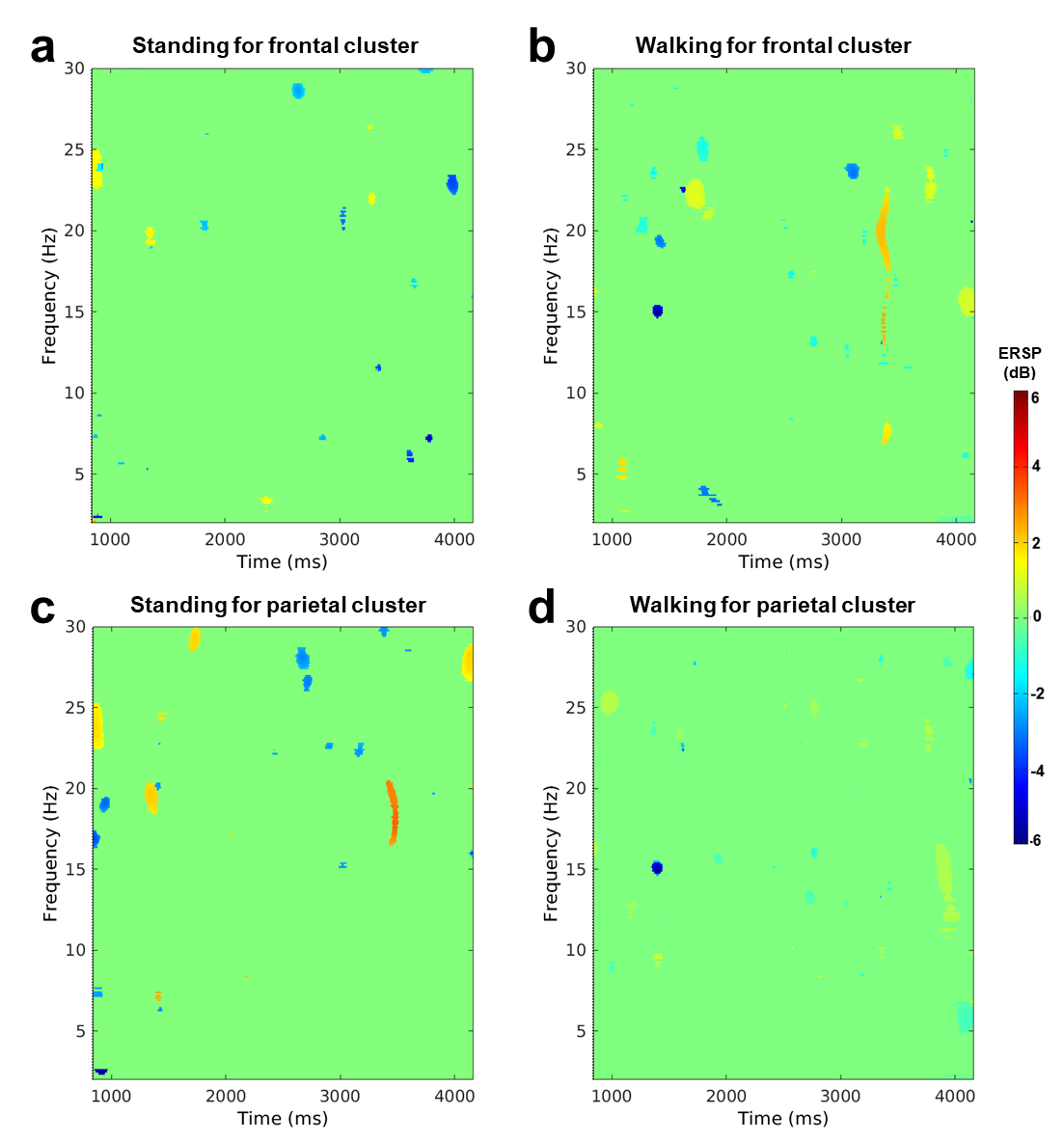
**

**Supplementary Figure 4: ERSPs in frontal and parietal clusters for baseline standing and walking.** (a-b) ERSPs in frontal cluster for baseline standing (a) and walking (b). (c-d) ERSPs in parietal cluster for baseline standing (c) and walking (d). For all ERSPs, non-significant points were masked with zero values and are displayed in green. Significant differences are displayed in red and blue for positive and negative deviations, respectively with *p* < .001.

*EEG processing pipeline.* The processing pipeline for EEG analysis is shown in Figure S5.


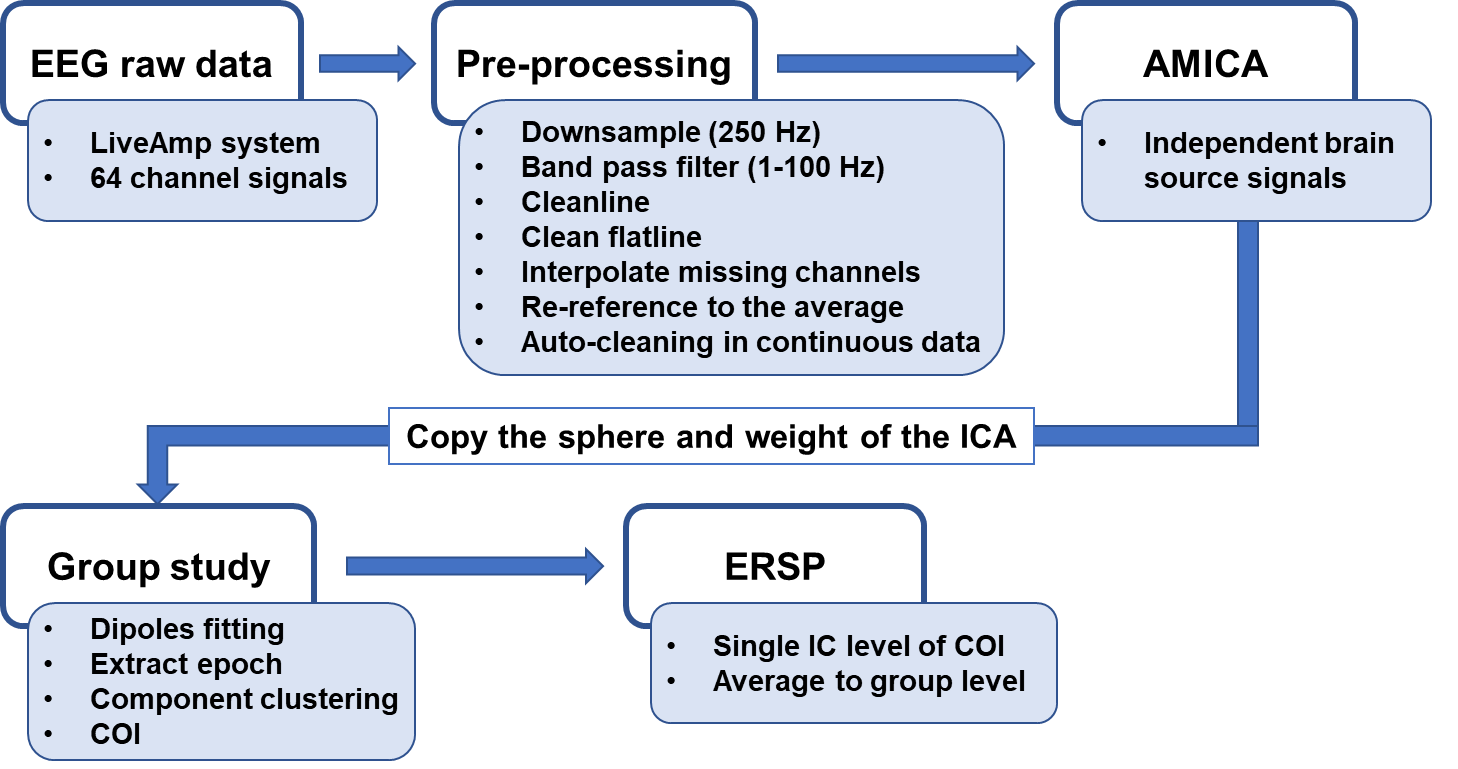


**Supplementary Figure 5 EEG preprocessing pipeline**

## **Supplementary Tables**

| **Supplementary Table 1: The post hoc comparison results for trial conditions (non-VGL and VGL)** | | | | | | | |
| --- | --- | --- | --- | --- | --- | --- | --- |
| landmark number | trial condition | | Mean Difference (nonVGL-VGL) | Std. Error | Sig. | 95% Confidence Interval for Difference | |
|  |  |  |  |  |  | Lower Bound | Upper Bound |
| 1 | non-VGL | VGL | 3.615^*^ | 1.407 | 0.013 | 0.790 | 6.441 |
| 2 | non-VGL | VGL | 6.154^*^ | 2.401 | 0.013 | 1.332 | 10.976 |
| 3 | non-VGL | VGL | 12.115^*^ | 1.348 | 0.000 | 9.408 | 14.823 |
| 4 | non-VGL | VGL | 8.577^*^ | 2.237 | 0.000 | 4.084 | 13.069 |
| 5 | non-VGL | VGL | 15.962^*^ | 2.569 | 0.000 | 10.801 | 21.122 |
| 6 | non-VGL | VGL | 4.154^*^ | 1.692 | 0.018 | 0.755 | 7.553 |
| 7 | non-VGL | VGL | 22.731^*^ | 4.963 | 0.000 | 12.762 | 32.699 |
| 8 | non-VGL | VGL | 7.462^*^ | 1.679 | 0.000 | 4.088 | 10.835 |
| 9 | non-VGL | VGL | 10.308^*^ | 1.712 | 0.000 | 6.870 | 13.746 |
| 10 | non-VGL | VGL | 14.192^*^ | 1.807 | 0.000 | 10.562 | 17.823 |
| 11 | non-VGL | VGL | 0.115 | 1.411 | 0.935 | -2.719 | 2.950 |
| * The mean difference is significant at the .05 level. | | | | | | | |

| **Supplementary Table 2: The post hoc comparison results for local landmarks** | | | | | | | |
| --- | --- | --- | --- | --- | --- | --- | --- |
| trial condition | landmark number | | Mean Difference (nonVGL-VGL) | Std. Error | Sig. | 95% Confidence Interval | |
|  |  |  |  |  |  | Lower Bound | Upper Bound |
| non-VGL | 1 | 2 | -8.00^*^ | 2.918 | 0.007 | -13.75 | -2.25 |
|  |  | 3 | -3.42 | 2.918 | 0.242 | -9.17 | 2.32 |
|  |  | 4 | 1.69 | 2.918 | 0.562 | -4.05 | 7.44 |
|  |  | 5 | -13.96^*^ | 2.918 | 0.000 | -19.71 | -8.22 |
|  |  | 6 | -1.62 | 2.918 | 0.580 | -7.36 | 4.13 |
|  |  | 7 | -21.92^*^ | 2.918 | 0.000 | -27.67 | -16.18 |
|  |  | 8 | -1.38 | 2.918 | 0.636 | -7.13 | 4.36 |
|  |  | 9 | -6.00^*^ | 2.918 | 0.041 | -11.75 | -0.25 |
|  |  | 10 | -9.65^*^ | 2.918 | 0.001 | -15.40 | -3.91 |
|  |  | 11 | 1.73 | 2.918 | 0.554 | -4.01 | 7.48 |
|  | 2 | 1 | 8.00^*^ | 2.918 | 0.007 | 2.25 | 13.75 |
|  |  | 3 | 4.58 | 2.918 | 0.118 | -1.17 | 10.32 |
|  |  | 4 | 9.69^*^ | 2.918 | 0.001 | 3.95 | 15.44 |
|  |  | 5 | -5.96^*^ | 2.918 | 0.042 | -11.71 | -0.22 |
|  |  | 6 | 6.38^*^ | 2.918 | 0.030 | 0.64 | 12.13 |
|  |  | 7 | -13.92^*^ | 2.918 | 0.000 | -19.67 | -8.18 |
|  |  | 8 | 6.62^*^ | 2.918 | 0.024 | 0.87 | 12.36 |
|  |  | 9 | 2.00 | 2.918 | 0.494 | -3.75 | 7.75 |
|  |  | 10 | -1.65 | 2.918 | 0.571 | -7.40 | 4.09 |
|  |  | 11 | 9.73^*^ | 2.918 | 0.001 | 3.99 | 15.48 |
|  | 3 | 1 | 3.42 | 2.918 | 0.242 | -2.32 | 9.17 |
|  |  | 2 | -4.58 | 2.918 | 0.118 | -10.32 | 1.17 |
|  |  | 4 | 5.12 | 2.918 | 0.081 | -0.63 | 10.86 |
|  |  | 5 | -10.54^*^ | 2.918 | 0.000 | -16.28 | -4.79 |
|  |  | 6 | 1.81 | 2.918 | 0.536 | -3.94 | 7.55 |
|  |  | 7 | -18.50^*^ | 2.918 | 0.000 | -24.25 | -12.75 |
|  |  | 8 | 2.04 | 2.918 | 0.485 | -3.71 | 7.78 |
|  |  | 9 | -2.58 | 2.918 | 0.378 | -8.32 | 3.17 |
|  |  | 10 | -6.23^*^ | 2.918 | 0.034 | -11.98 | -0.49 |
|  |  | 11 | 5.15 | 2.918 | 0.079 | -0.59 | 10.90 |
|  | 4 | 1 | -1.69 | 2.918 | 0.562 | -7.44 | 4.05 |
|  |  | 2 | -9.69^*^ | 2.918 | 0.001 | -15.44 | -3.95 |
|  |  | 3 | -5.12 | 2.918 | 0.081 | -10.86 | 0.63 |
|  |  | 5 | -15.65^*^ | 2.918 | 0.000 | -21.40 | -9.91 |
|  |  | 6 | -3.31 | 2.918 | 0.258 | -9.05 | 2.44 |
|  |  | 7 | -23.62^*^ | 2.918 | 0.000 | -29.36 | -17.87 |
|  |  | 8 | -3.08 | 2.918 | 0.293 | -8.82 | 2.67 |
|  |  | 9 | -7.69^*^ | 2.918 | 0.009 | -13.44 | -1.95 |
|  |  | 10 | -11.35^*^ | 2.918 | 0.000 | -17.09 | -5.60 |
|  |  | 11 | 0.04 | 2.918 | 0.989 | -5.71 | 5.78 |
|  | 5 | 1 | 13.96^*^ | 2.918 | 0.000 | 8.22 | 19.71 |
|  |  | 2 | 5.96^*^ | 2.918 | 0.042 | 0.22 | 11.71 |
|  |  | 3 | 10.54^*^ | 2.918 | 0.000 | 4.79 | 16.28 |
|  |  | 4 | 15.65^*^ | 2.918 | 0.000 | 9.91 | 21.40 |
|  |  | 6 | 12.35^*^ | 2.918 | 0.000 | 6.60 | 18.09 |
|  |  | 7 | -7.96^*^ | 2.918 | 0.007 | -13.71 | -2.22 |
|  |  | 8 | 12.58^*^ | 2.918 | 0.000 | 6.83 | 18.32 |
|  |  | 9 | 7.96^*^ | 2.918 | 0.007 | 2.22 | 13.71 |
|  |  | 10 | 4.31 | 2.918 | 0.141 | -1.44 | 10.05 |
|  |  | 11 | 15.69^*^ | 2.918 | 0.000 | 9.95 | 21.44 |
|  | 6 | 1 | 1.62 | 2.918 | 0.580 | -4.13 | 7.36 |
|  |  | 2 | -6.38^*^ | 2.918 | 0.030 | -12.13 | -0.64 |
|  |  | 3 | -1.81 | 2.918 | 0.536 | -7.55 | 3.94 |
|  |  | 4 | 3.31 | 2.918 | 0.258 | -2.44 | 9.05 |
|  |  | 5 | -12.35^*^ | 2.918 | 0.000 | -18.09 | -6.60 |
|  |  | 7 | -20.31^*^ | 2.918 | 0.000 | -26.05 | -14.56 |
|  |  | 8 | 0.23 | 2.918 | 0.937 | -5.51 | 5.98 |
|  |  | 9 | -4.38 | 2.918 | 0.134 | -10.13 | 1.36 |
|  |  | 10 | -8.04^*^ | 2.918 | 0.006 | -13.78 | -2.29 |
|  |  | 11 | 3.35 | 2.918 | 0.253 | -2.40 | 9.09 |
|  | 7 | 1 | 21.92^*^ | 2.918 | 0.000 | 16.18 | 27.67 |
|  |  | 2 | 13.92^*^ | 2.918 | 0.000 | 8.18 | 19.67 |
|  |  | 3 | 18.50^*^ | 2.918 | 0.000 | 12.75 | 24.25 |
|  |  | 4 | 23.62^*^ | 2.918 | 0.000 | 17.87 | 29.36 |
|  |  | 5 | 7.96^*^ | 2.918 | 0.007 | 2.22 | 13.71 |
|  |  | 6 | 20.31^*^ | 2.918 | 0.000 | 14.56 | 26.05 |
|  |  | 8 | 20.54^*^ | 2.918 | 0.000 | 14.79 | 26.28 |
|  |  | 9 | 15.92^*^ | 2.918 | 0.000 | 10.18 | 21.67 |
|  |  | 10 | 12.27^*^ | 2.918 | 0.000 | 6.52 | 18.01 |
|  |  | 11 | 23.65^*^ | 2.918 | 0.000 | 17.91 | 29.40 |
|  | 8 | 1 | 1.38 | 2.918 | 0.636 | -4.36 | 7.13 |
|  |  | 2 | -6.62^*^ | 2.918 | 0.024 | -12.36 | -0.87 |
|  |  | 3 | -2.04 | 2.918 | 0.485 | -7.78 | 3.71 |
|  |  | 4 | 3.08 | 2.918 | 0.293 | -2.67 | 8.82 |
|  |  | 5 | -12.58^*^ | 2.918 | 0.000 | -18.32 | -6.83 |
|  |  | 6 | -0.23 | 2.918 | 0.937 | -5.98 | 5.51 |
|  |  | 7 | -20.54^*^ | 2.918 | 0.000 | -26.28 | -14.79 |
|  |  | 9 | -4.62 | 2.918 | 0.115 | -10.36 | 1.13 |
|  |  | 10 | -8.27^*^ | 2.918 | 0.005 | -14.01 | -2.52 |
|  |  | 11 | 3.12 | 2.918 | 0.287 | -2.63 | 8.86 |
|  | 9 | 1 | 6.00^*^ | 2.918 | 0.041 | 0.25 | 11.75 |
|  |  | 2 | -2.00 | 2.918 | 0.494 | -7.75 | 3.75 |
|  |  | 3 | 2.58 | 2.918 | 0.378 | -3.17 | 8.32 |
|  |  | 4 | 7.69^*^ | 2.918 | 0.009 | 1.95 | 13.44 |
|  |  | 5 | -7.96^*^ | 2.918 | 0.007 | -13.71 | -2.22 |
|  |  | 6 | 4.38 | 2.918 | 0.134 | -1.36 | 10.13 |
|  |  | 7 | -15.92^*^ | 2.918 | 0.000 | -21.67 | -10.18 |
|  |  | 8 | 4.62 | 2.918 | 0.115 | -1.13 | 10.36 |
|  |  | 10 | -3.65 | 2.918 | 0.212 | -9.40 | 2.09 |
|  |  | 11 | 7.73^*^ | 2.918 | 0.009 | 1.99 | 13.48 |
|  | 10 | 1 | 9.65^*^ | 2.918 | 0.001 | 3.91 | 15.40 |
|  |  | 2 | 1.65 | 2.918 | 0.571 | -4.09 | 7.40 |
|  |  | 3 | 6.23^*^ | 2.918 | 0.034 | 0.49 | 11.98 |
|  |  | 4 | 11.35^*^ | 2.918 | 0.000 | 5.60 | 17.09 |
|  |  | 5 | -4.31 | 2.918 | 0.141 | -10.05 | 1.44 |
|  |  | 6 | 8.04^*^ | 2.918 | 0.006 | 2.29 | 13.78 |
|  |  | 7 | -12.27^*^ | 2.918 | 0.000 | -18.01 | -6.52 |
|  |  | 8 | 8.27^*^ | 2.918 | 0.005 | 2.52 | 14.01 |
|  |  | 9 | 3.65 | 2.918 | 0.212 | -2.09 | 9.40 |
|  |  | 11 | 11.38^*^ | 2.918 | 0.000 | 5.64 | 17.13 |
|  | 11 | 1 | -1.73 | 2.918 | 0.554 | -7.48 | 4.01 |
|  |  | 2 | -9.73^*^ | 2.918 | 0.001 | -15.48 | -3.99 |
|  |  | 3 | -5.15 | 2.918 | 0.079 | -10.90 | 0.59 |
|  |  | 4 | -0.04 | 2.918 | 0.989 | -5.78 | 5.71 |
|  |  | 5 | -15.69^*^ | 2.918 | 0.000 | -21.44 | -9.95 |
|  |  | 6 | -3.35 | 2.918 | 0.253 | -9.09 | 2.40 |
|  |  | 7 | -23.65^*^ | 2.918 | 0.000 | -29.40 | -17.91 |
|  |  | 8 | -3.12 | 2.918 | 0.287 | -8.86 | 2.63 |
|  |  | 9 | -7.73^*^ | 2.918 | 0.009 | -13.48 | -1.99 |
|  |  | 10 | -11.38^*^ | 2.918 | 0.000 | -17.13 | -5.64 |
| VGL | 1 | 2 | -5.46^*^ | 1.548 | 0.000 | -8.51 | -2.42 |
|  |  | 3 | 5.08^*^ | 1.548 | 0.001 | 2.03 | 8.12 |
|  |  | 4 | -9.42^*^ | 1.548 | 0.000 | -12.47 | -6.38 |
|  |  | 5 | -1.62 | 1.548 | 0.297 | -4.66 | 1.43 |
|  |  | 6 | -1.08 | 1.548 | 0.487 | -4.12 | 1.97 |
|  |  | 7 | -2.81 | 1.548 | 0.071 | -5.85 | 0.24 |
|  |  | 8 | 2.46 | 1.548 | 0.113 | -0.58 | 5.51 |
|  |  | 9 | 0.69 | 1.548 | 0.655 | -2.35 | 3.74 |
|  |  | 10 | 0.92 | 1.548 | 0.551 | -2.12 | 3.97 |
|  |  | 11 | -1.77 | 1.548 | 0.254 | -4.82 | 1.28 |
|  | 2 | 1 | 5.46^*^ | 1.548 | 0.000 | 2.42 | 8.51 |
|  |  | 3 | 10.54^*^ | 1.548 | 0.000 | 7.49 | 13.58 |
|  |  | 4 | -3.96^*^ | 1.548 | 0.011 | -7.01 | -0.92 |
|  |  | 5 | 3.85^*^ | 1.548 | 0.014 | 0.80 | 6.89 |
|  |  | 6 | 4.38^*^ | 1.548 | 0.005 | 1.34 | 7.43 |
|  |  | 7 | 2.65 | 1.548 | 0.087 | -0.39 | 5.70 |
|  |  | 8 | 7.92^*^ | 1.548 | 0.000 | 4.88 | 10.97 |
|  |  | 9 | 6.15^*^ | 1.548 | 0.000 | 3.11 | 9.20 |
|  |  | 10 | 6.38^*^ | 1.548 | 0.000 | 3.34 | 9.43 |
|  |  | 11 | 3.69^*^ | 1.548 | 0.018 | 0.65 | 6.74 |
|  | 3 | 1 | -5.08^*^ | 1.548 | 0.001 | -8.12 | -2.03 |
|  |  | 2 | -10.54^*^ | 1.548 | 0.000 | -13.58 | -7.49 |
|  |  | 4 | -14.50^*^ | 1.548 | 0.000 | -17.55 | -11.45 |
|  |  | 5 | -6.69^*^ | 1.548 | 0.000 | -9.74 | -3.65 |
|  |  | 6 | -6.15^*^ | 1.548 | 0.000 | -9.20 | -3.11 |
|  |  | 7 | -7.88^*^ | 1.548 | 0.000 | -10.93 | -4.84 |
|  |  | 8 | -2.62 | 1.548 | 0.092 | -5.66 | 0.43 |
|  |  | 9 | -4.38^*^ | 1.548 | 0.005 | -7.43 | -1.34 |
|  |  | 10 | -4.15^*^ | 1.548 | 0.008 | -7.20 | -1.11 |
|  |  | 11 | -6.85^*^ | 1.548 | 0.000 | -9.89 | -3.80 |
|  | 4 | 1 | 9.42^*^ | 1.548 | 0.000 | 6.38 | 12.47 |
|  |  | 2 | 3.96^*^ | 1.548 | 0.011 | 0.92 | 7.01 |
|  |  | 3 | 14.50^*^ | 1.548 | 0.000 | 11.45 | 17.55 |
|  |  | 5 | 7.81^*^ | 1.548 | 0.000 | 4.76 | 10.85 |
|  |  | 6 | 8.35^*^ | 1.548 | 0.000 | 5.30 | 11.39 |
|  |  | 7 | 6.62^*^ | 1.548 | 0.000 | 3.57 | 9.66 |
|  |  | 8 | 11.88^*^ | 1.548 | 0.000 | 8.84 | 14.93 |
|  |  | 9 | 10.12^*^ | 1.548 | 0.000 | 7.07 | 13.16 |
|  |  | 10 | 10.35^*^ | 1.548 | 0.000 | 7.30 | 13.39 |
|  |  | 11 | 7.65^*^ | 1.548 | 0.000 | 4.61 | 10.70 |
|  | 5 | 1 | 1.62 | 1.548 | 0.297 | -1.43 | 4.66 |
|  |  | 2 | -3.85^*^ | 1.548 | 0.014 | -6.89 | -0.80 |
|  |  | 3 | 6.69^*^ | 1.548 | 0.000 | 3.65 | 9.74 |
|  |  | 4 | -7.81^*^ | 1.548 | 0.000 | -10.85 | -4.76 |
|  |  | 6 | 0.54 | 1.548 | 0.728 | -2.51 | 3.58 |
|  |  | 7 | -1.19 | 1.548 | 0.442 | -4.24 | 1.85 |
|  |  | 8 | 4.08^*^ | 1.548 | 0.009 | 1.03 | 7.12 |
|  |  | 9 | 2.31 | 1.548 | 0.137 | -0.74 | 5.35 |
|  |  | 10 | 2.54 | 1.548 | 0.102 | -0.51 | 5.58 |
|  |  | 11 | -0.15 | 1.548 | 0.921 | -3.20 | 2.89 |
|  | 6 | 1 | 1.08 | 1.548 | 0.487 | -1.97 | 4.12 |
|  |  | 2 | -4.38^*^ | 1.548 | 0.005 | -7.43 | -1.34 |
|  |  | 3 | 6.15^*^ | 1.548 | 0.000 | 3.11 | 9.20 |
|  |  | 4 | -8.35^*^ | 1.548 | 0.000 | -11.39 | -5.30 |
|  |  | 5 | -0.54 | 1.548 | 0.728 | -3.58 | 2.51 |
|  |  | 7 | -1.73 | 1.548 | 0.264 | -4.78 | 1.32 |
|  |  | 8 | 3.54^*^ | 1.548 | 0.023 | 0.49 | 6.58 |
|  |  | 9 | 1.77 | 1.548 | 0.254 | -1.28 | 4.82 |
|  |  | 10 | 2.00 | 1.548 | 0.197 | -1.05 | 5.05 |
|  |  | 11 | -0.69 | 1.548 | 0.655 | -3.74 | 2.35 |
|  | 7 | 1 | 2.81 | 1.548 | 0.071 | -0.24 | 5.85 |
|  |  | 2 | -2.65 | 1.548 | 0.087 | -5.70 | 0.39 |
|  |  | 3 | 7.88^*^ | 1.548 | 0.000 | 4.84 | 10.93 |
|  |  | 4 | -6.62^*^ | 1.548 | 0.000 | -9.66 | -3.57 |
|  |  | 5 | 1.19 | 1.548 | 0.442 | -1.85 | 4.24 |
|  |  | 6 | 1.73 | 1.548 | 0.264 | -1.32 | 4.78 |
|  |  | 8 | 5.27^*^ | 1.548 | 0.001 | 2.22 | 8.32 |
|  |  | 9 | 3.50^*^ | 1.548 | 0.024 | 0.45 | 6.55 |
|  |  | 10 | 3.73^*^ | 1.548 | 0.017 | 0.68 | 6.78 |
|  |  | 11 | 1.04 | 1.548 | 0.503 | -2.01 | 4.08 |
|  | 8 | 1 | -2.46 | 1.548 | 0.113 | -5.51 | 0.58 |
|  |  | 2 | -7.92^*^ | 1.548 | 0.000 | -10.97 | -4.88 |
|  |  | 3 | 2.62 | 1.548 | 0.092 | -0.43 | 5.66 |
|  |  | 4 | -11.88^*^ | 1.548 | 0.000 | -14.93 | -8.84 |
|  |  | 5 | -4.08^*^ | 1.548 | 0.009 | -7.12 | -1.03 |
|  |  | 6 | -3.54^*^ | 1.548 | 0.023 | -6.58 | -0.49 |
|  |  | 7 | -5.27^*^ | 1.548 | 0.001 | -8.32 | -2.22 |
|  |  | 9 | -1.77 | 1.548 | 0.254 | -4.82 | 1.28 |
|  |  | 10 | -1.54 | 1.548 | 0.321 | -4.58 | 1.51 |
|  |  | 11 | -4.23^*^ | 1.548 | 0.007 | -7.28 | -1.18 |
|  | 9 | 1 | -0.69 | 1.548 | 0.655 | -3.74 | 2.35 |
|  |  | 2 | -6.15^*^ | 1.548 | 0.000 | -9.20 | -3.11 |
|  |  | 3 | 4.38^*^ | 1.548 | 0.005 | 1.34 | 7.43 |
|  |  | 4 | -10.12^*^ | 1.548 | 0.000 | -13.16 | -7.07 |
|  |  | 5 | -2.31 | 1.548 | 0.137 | -5.35 | 0.74 |
|  |  | 6 | -1.77 | 1.548 | 0.254 | -4.82 | 1.28 |
|  |  | 7 | -3.50^*^ | 1.548 | 0.024 | -6.55 | -0.45 |
|  |  | 8 | 1.77 | 1.548 | 0.254 | -1.28 | 4.82 |
|  |  | 10 | 0.23 | 1.548 | 0.882 | -2.82 | 3.28 |
|  |  | 11 | -2.46 | 1.548 | 0.113 | -5.51 | 0.58 |
|  | 10 | 1 | -0.92 | 1.548 | 0.551 | -3.97 | 2.12 |
|  |  | 2 | -6.38^*^ | 1.548 | 0.000 | -9.43 | -3.34 |
|  |  | 3 | 4.15^*^ | 1.548 | 0.008 | 1.11 | 7.20 |
|  |  | 4 | -10.35^*^ | 1.548 | 0.000 | -13.39 | -7.30 |
|  |  | 5 | -2.54 | 1.548 | 0.102 | -5.58 | 0.51 |
|  |  | 6 | -2.00 | 1.548 | 0.197 | -5.05 | 1.05 |
|  |  | 7 | -3.73^*^ | 1.548 | 0.017 | -6.78 | -0.68 |
|  |  | 8 | 1.54 | 1.548 | 0.321 | -1.51 | 4.58 |
|  |  | 9 | -0.23 | 1.548 | 0.882 | -3.28 | 2.82 |
|  |  | 11 | -2.69 | 1.548 | 0.083 | -5.74 | 0.35 |
|  | 11 | 1 | 1.77 | 1.548 | 0.254 | -1.28 | 4.82 |
|  |  | 2 | -3.69^*^ | 1.548 | 0.018 | -6.74 | -0.65 |
|  |  | 3 | 6.85^*^ | 1.548 | 0.000 | 3.80 | 9.89 |
|  |  | 4 | -7.65^*^ | 1.548 | 0.000 | -10.70 | -4.61 |
|  |  | 5 | 0.15 | 1.548 | 0.921 | -2.89 | 3.20 |
|  |  | 6 | 0.69 | 1.548 | 0.655 | -2.35 | 3.74 |
|  |  | 7 | -1.04 | 1.548 | 0.503 | -4.08 | 2.01 |
|  |  | 8 | 4.23^*^ | 1.548 | 0.007 | 1.18 | 7.28 |
|  |  | 9 | 2.46 | 1.548 | 0.113 | -0.58 | 5.51 |
|  |  | 10 | 2.69 | 1.548 | 0.083 | -0.35 | 5.74 |
| * The mean difference is significant at the .05 level. | | | | | | | |

## **Supplementary Methods**

*The effects results of gender as the between-subjects factor of ANOVAs.* While analyzing one-way repeated-measures ANOVAs for our variables presented in results section of our manuscript, gender was added as a between-subjects factor, however, there is no significant interaction between gender and trial conditions (non-VGL and VGL trials) for all variables. The test results of between-subjects effects are: (a) Average response time in pointing task - F1,25=.308, p=.584, partial η2=.012; (b) Average angular error in pointing task - F1,25=1.232, p=.278, partial η2=.047; (c) Average response time in wayfinding task - F1,25=.208, p=.652, partial η2=.008; (d) Average distance traveled in wayfinding task - F1,25=.407, p=.529, partial η2=.016; (e) Average left pupil diameter - F1,24=.022, p=.883, partial η2=.001; and (f) Average right pupil diameter - F1,24=.000, p=.985, partial η2=.000.

*The post hoc comparison results of trial type and landmarks for local landmark fixations in non-VGL and VGL trials.* This is the post hoc analysis for fixation on local landmarks. The results for trial conditions (non-VGL and VGL) are presented in Table S1 of excel file (Supplementary Dataset 1.xlsx) and for local landmarks (from landmark 1 to landmark 11) presented in Table S2 of excel file (Supplementary Dataset 1.xlsx)

*EEG pre-processing pipeline code.* This code script is used for EEG pre-processing based on EEGLAB toolbox version 2020.0 with MATLAB version 2018a (MathWorks Inc., USA).

%%

% Author: Jia Liu

% Australian Artificial Intelligence Institute, University of Technology Sydney (UTS)

% Email: Jia.Liu@uts.edu.au

%%

% This script for preprocessing EEG data

% It can be used for general EEG data experiment

% The script can be adapted with different methods and parameters in each steps for suitable with the experiment design

%% General information

eeglabDir = '/data/software/eeglab_2020/'; % the path of EEGLAB toolbox

addpath(eeglabDir);

ALLparticipantID = {'01', '02', '03', '04', '05', '06','07','08','09','10','11',...

'12','13','14','15','16','17','18','19','20','21','22','23','24','25','26','27'};

for number = 1:size(ALLparticipantID,2)

clearvars -except ALLparticipantID number eeglabDir

close all

clc

%% General information

ParticipantID = ALLparticipantID{number};

EEGFileName = sprintf('%s%s','S',ParticipantID);

EEGFileName1 = sprintf('%s%s%s','S',ParticipantID,'_baselineExploration'); % EEG raw dataset of baseline and exploration sections

EEGFileName2 = sprintf('%s%s%s','S',ParticipantID,'_wayfindingPointing'); % EEG raw dataset of wayfinding and pointing sections

dataDir = sprintf('%s%s%s','/data/rawdata_EEG/S',ParticipantID,'/'); % EEG raw data folder

dataCleanDir = sprintf('%s%s%s','/data/DataClean/'); % EEG cleaned data folder (cleaned data will be saved here)

%% Create the subfolder to store the preprocessing datasets

if ~exist(dataCleanDir)

mkdir(dataCleanDir);end

%% Structure of the pipeline

% Step 1: Start EEGLAB

% Step 2: Load EEG data into eeglab

% Step 3: Filtering [1 100] and Downsample to 250Hz for each task dataset.

% Step 3: Merge multiple datasets

% Step 4: Band-pass filter.

% Step 5: Import channel info

% Step 6: Remove line noise using CleanLine

% Step 7: Clean flatlines

% Step 8: Clean noise channels

% Step 9: Check the continuous data quality

% Step 10: Interpolate the missing channels

% Step 11: Re-reference

% Step 12: (Optional) Check the continuous data quality 1 more time

% Step 13: Run the ICA with AMICA

% Step 14: Estimate single equivalent current dipoles

% Step 15: Save the cleaned dataset with AMICA solution

% Step 16: Visualize the AMICA solution

%% The program starts from here

% Step 1: Start your eeglab library

[ALLEEG EEG CURRENTSET ALLCOM] = eeglab;close;

dipfitdefs;

display(['Processing the dataset ' EEGFileName]);

nameDataCleaned = [EEGFileName '_CleanedAMICA'];

% Step 2: Loading your dataset, it depends on your data type, here is *.set file

EEG1 = pop_loadset('filename', [EEGFileName1 '.set'], 'filepath', dataDir);

EEG2 = pop_loadset('filename', [EEGFileName2 '.set'], 'filepath', dataDir);

% Step 3: Filtering [1 100] and Downsample to 250Hz for each task dataset.

EEG1 = pop_eegfiltnew(EEG1,[],100);

EEG1 = pop_eegfiltnew(EEG1,1,[]);

EEG1 = pop_resample(EEG1, 250);

EEG2 = pop_eegfiltnew(EEG2,[],100);

EEG2 = pop_eegfiltnew(EEG2,1,[]);

EEG2 = pop_resample(EEG2, 250);

% Step 4: Merge datasets of baselineExploration and pointing&wayfinding

EEG=pop_mergeset( EEG1, EEG2);

% Step 5: Import channel info

ChanLocsLabels = {'Fp1' 'Fz' 'F3' 'F7' 'FT9' 'FC5' 'FC1' 'C3' 'T7' 'TP9' 'CP5' 'CP1' 'Pz' 'P3' 'P7'...

'O1' 'Oz' 'O2' 'P4' 'P8' 'TP10' 'CP6' 'CP2' 'Cz' 'C4' 'T8' 'FT10' 'FC6' 'FC2' 'F4'...

'F8' 'Fp2' 'AF7' 'AF3' 'AFz' 'F1' 'F5' 'FT7' 'FC3' 'C1' 'C5' 'TP7' 'CP3' 'P1' 'P5'...

'PO7' 'PO3' 'POz' 'PO4' 'PO8' 'P6' 'P2' 'CPz' 'CP4' 'TP8' 'C6' 'C2' 'FC4' 'FT8' 'F6'...

'AF8' 'AF4' 'F2' 'Iz'};

for numChans = 1:length(EEG.chanlocs)

EEG.chanlocs(numChans).labels = ChanLocsLabels{numChans} ;

end

EEG = pop_chanedit(EEG, 'lookup',[eeglabDir 'plugins/dipfit3.7/standard_BEM/elec/standard_1005.elc'],'eval','chans = pop_chancenter( chans, [],[]);');

% Step 6: Remove line noise using CleanLine

EEG = pop_cleanline(EEG, 'bandwidth', 2,'chanlist', [1:EEG.nbchan], 'computepower', 0, 'linefreqs', [50 100 150 200 250],...

'normSpectrum', 0, 'p', 0.01, 'pad', 2, 'plotfigures', 0, 'scanforlines', 1, 'sigtype', 'Channels', 'tau', 100,...

'verb', 1, 'winsize', 4, 'winstep', 4);

EEG = eeg_checkset(EEG);

% Step 7: Clean flatlines

EEG = clean_flatlines(EEG,5); % 5s lost data

% Step 8: Clean noise channels

EEG = clean_channels(EEG);

% Step 9: Check the continuous data quality

EEG = pop_rejcont(EEG, 'elecrange', [1:size(EEG.chanlocs,2)],...

'freqlimit', [1 100], 'threshold', 10, 'epochlength', 0.5,...

'contiguous', 4, 'addlength', 0.25, 'taper', 'hamming'); % continous removal here

% Step 10: Interpolate the missing channels

EEG = pop_interp(EEG, originalEEG.chanlocs, 'spherical');

% Step 11: Re-reference

EEG = pop_reref(EEG, []);

EEG = eeg_checkset(EEG);

% Step 12: Check the continuous data quality 1 more time

EEG = pop_rejcont(EEG, 'elecrange', [1:size(EEG.chanlocs,2)],...

'freqlimit', [1 100], 'threshold', 10, 'epochlength', 0.5,...

'contiguous', 8, 'addlength', 0.25, 'taper', 'hamming'); % continous removal here

% Step 13: Run ICA with AMICA

runamica15(EEG.data, 'num_chans', EEG.nbchan,...

'outdir', [dataCleanDir nameDataCleaned],...

'num_models', 1, 'do_reject', 1, 'numrej', 15, 'rejsig', 3, 'rejint', 1);

EEG.etc.amica = loadmodout15([dataCleanDir nameDataCleaned]);

EEG.icaweights = EEG.etc.amica.W;

EEG.icasphere = EEG.etc.amica.S;

disp('runica successfull, storing weights and sphere.');

EEG = eeg_checkset(EEG, 'ica');

numICs = size(EEG.icaweights,1);

% Step 15: Estimate single equivalent current dipoles

[newlocs coordinateTransformParameters] = coregister(EEG.chanlocs, template_models(2).chanfile, 'warp', 'auto', 'manual', 'off');

templateChannelFilePath = [eeglabDir 'plugins/dipfit3.7/standard_BEM/elec/standard_1005.elc'];

hdmFilePath = [eeglabDir 'plugins/dipfit3.7/standard_BEM/standard_vol.mat'];

EEG = pop_dipfit_settings( EEG, 'hdmfile', hdmFilePath, 'coordformat', 'MNI',...

'mrifile', [eeglabDir 'plugins/dipfit3.7/standard_BEM/standard_mri.mat'],...

'chanfile', templateChannelFilePath, 'coord_transform', coordinateTransformParameters,...

'chansel', 1:EEG.nbchan);

EEG = pop_multifit(EEG, 1:EEG.nbchan,'threshold', 100, 'dipplot','off','plotopt',{'normlen' 'on'});

EEG = eeg_checkset(EEG);

% Step 16: Save the cleaned dataset with ICA solution

EEG = pop_saveset( EEG, 'filename', nameDataCleaned, 'filepath', dataCleanDir);

% Step 17: Visualize the ICA solution

pop_topoplot(EEG,0, [1:numICs] ,[nameDataCleaned '_AMICA'],[] ,1,'electrodes','off');

print([dataCleanDir nameDataCleaned '_AMICA.png'],'-dpng');

end

## **Supplementary References**

1. Cao Y. Bivariant Kernel Density Estimation. 2.1 edn. MATLAB (2021).
