# Supplementary material for: Redesigning navigational aids using virtual global landmarks to improve spatial knowledge retrieval
Source: NPJ Sci Learn. 2022 Jul 19;7:17. doi: 10.1038/s41539-022-00132-z (PMC9296625; doi:10.1038/s41539-022-00132-z)
Supplement: Supplementary file 2 — Reporting Summary [file 41539_2022_132_MOESM2_ESM.pdf]

nature portfolio

Corresponding author(s): Avinash K. Singh

Last updated by author(s): Dec 9, 2021

Reporting Summary

Nature Portfolio wishes to improve the reproducibility of the work that we publish. This form provides structure for consistency and transparency in reporting. For further information on Nature Portfolio policies, see our Editorial Policies and the Editorial Policy Checklist.

Statistics

For all statistical analyses, confirm that the following items are present in the figure legend, table legend, main text, or Methods section.

n/a	Confirmed	
☐	☒	The exact sample size (*n*) for each experimental group/condition, given as a discrete number and unit of measurement
☐	☒	A statement on whether measurements were taken from distinct samples or whether the same sample was measured repeatedly
☐	☒	The statistical test(s) used AND whether they are one- or two-sided<br>*Only common tests should be described solely by name; describe more complex techniques in the Methods section.*
☐	☒	A description of all covariates tested
☐	☒	A description of any assumptions or corrections, such as tests of normality and adjustment for multiple comparisons
☐	☒	A full description of the statistical parameters including central tendency (e.g. means) or other basic estimates (e.g. regression coefficient) AND variation (e.g. standard deviation) or associated estimates of uncertainty (e.g. confidence intervals)
☐	☒	For null hypothesis testing, the test statistic (e.g. *F*, *t*, *r*) with confidence intervals, effect sizes, degrees of freedom and *P* value noted<br>*Give P values as exact values whenever suitable.*
☒	☐	For Bayesian analysis, information on the choice of priors and Markov chain Monte Carlo settings
☒	☐	For hierarchical and complex designs, identification of the appropriate level for tests and full reporting of outcomes
☐	☒	Estimates of effect sizes (e.g. Cohen's *d*, Pearson's *r*), indicating how they were calculated

Our web collection on statistics for biologists contains articles on many of the points above.

Software and code

Policy information about availability of computer code

Data collection	The eye activity and behavior events were recorded with HTC's Vive Pro eye headset; the EEG data were recorded using Brain Vision's LiveAmp 64 system. All data streams from the EEG cap, eye tracker and head-mounted display were synchronized with Lab Streaming Layer (LSL).
Data analysis	Except brain data, all statistical analyses were conducted using SPSS Statistics 26. EEG data was analyzed using EEGLAB toolbox of MATLAB version as described in the manuscript.

For manuscripts utilizing custom algorithms or software that are central to the research but not yet described in published literature, software must be made available to editors and reviewers. We strongly encourage code deposition in a community repository (e.g. GitHub). See the Nature Portfolio guidelines for submitting code & software for further information.

Data

Policy information about availability of data

All manuscripts must include a data availability statement. This statement should provide the following information, where applicable:

- Accession codes, unique identifiers, or web links for publicly available datasets
- A description of any restrictions on data availability
- For clinical datasets or third party data, please ensure that the statement adheres to our policy

The data that support the findings of this study are available upon request from the corresponding author.

Field-specific reporting

Please select the one below that is the best fit for your research. If you are not sure, read the appropriate sections before making your selection.

☐ Life sciences ☒ Behavioural & social sciences ☐ Ecological, evolutionary & environmental sciences

For a reference copy of the document with all sections, see nature.com/documents/nr-reporting-summary-flat.pdf

Behavioural & social sciences study design

All studies must disclose on these points even when the disclosure is negative.

Study description	The study reports one experiment of mixed methods with quantitative behavior experiment and EEG-based neuroimaging.
Research sample	The experiment involved 27 participants: 9 females and 18 males, mean age: 28.19.
Sampling strategy	Because there is no standard method for estimating sample size for this type of study, we attempted to identify as many datasets as possible that linked navigational behavior and brain dynamics as a mobile brain/body imaging (MoBI) investigation approach. To our knowledge, this is the largest study of its kind.
Data collection	The experiment took place in an experimental area at the Tech Lab in University of Technology Sydney. We first conducted a pre-test on all participants to assess their individual spatial abilities before starting. The participants then explored the Sydney Park scenario along a predefined route as shown in Figure 5c in the manuscript. Afterwards, they performed two specific navigational tasks: one pointing task and one wayfinding task. All the tasks were conducted inside the Sydney Park scenario and involved active navigation, including physical walking. The eye activity and behavior events were recorded with HTC's Vive Pro eye headset; the EEG data were recorded using Brain Vision's LiveAmp 64 system. All data streams from the EEG cap, eye tracker and head-mounted display were synchronized with Lab Streaming Layer (LSL).
Timing	From 27 July 2020 to 23 September 2020.
Data exclusions	For EEG data, of the 27 participants, data for two were excluded due to discontinuous recording; for eye activity, one participant's dataset was failed to be recorded with eye tracker due to technical issue. During statistical analysis on behavior data, all outliers inspected by boxplots for values greater than 1.5 box lengths from the edge of the box were removed. For response time in pointing task, nine outliers out of 162 non-VGL target trials and eight outliers out of 162 VGL target trials were removed; for angular error in pointing task, five outliers out of 162 non-VGL target trials and five outliers out of 162 VGL target trials were removed; for response time in wayfinding task, seven outliers out of 162 non-VGL target trials and eleven outliers out of 162 VGL target trials were removed; for distance traveled in wayfinding task, eighteen outliers out of 162 non-VGL target trials and fourteen outliers out of 162 VGL target trials were removed.
Non-participation	For EEG data, two participants' datasets were excluded due to poor EEG quality (discontinuous recording); for eye activity, one participant's dataset was failed to be recorded with eye tracker due to technical issue.
Randomization	Participants were not allocated into any group.

Reporting for specific materials, systems and methods

We require information from authors about some types of materials, experimental systems and methods used in many studies. Here, indicate whether each material, system or method listed is relevant to your study. If you are not sure if a list item applies to your research, read the appropriate section before selecting a response.

Materials & experimental systems

n/a	Involved in the study	
☒	☐	Antibodies
☒	☐	Eukaryotic cell lines
☒	☐	Palaeontology and archaeology
☒	☐	Animals and other organisms
☐	☒	Human research participants
☒	☐	Clinical data
☒	☐	Dual use research of concern

Methods

n/a	Involved in the study	
☒	☐	ChIP-seq
☒	☐	Flow cytometry
☒	☐	MRI-based neuroimaging

Human research participants

Policy information about studies involving human research participants

Population characteristics	27 participants: 9 females and 18 males, mean age: 28.19.

Recruitment	They were randomly recruited through advertisements posted on social media. Informed consent was obtained from all the participants.
Ethics oversight	This study obtained the approval of the institute's human research ethics committee of University of Technology Sydney (UTS), Australia (Grant number: UTS HREC REF NO. ETH17-2095)

Note that full information on the approval of the study protocol must also be provided in the manuscript.
